# Epithelium-autonomous NAIP/NLRC4 prevents TNF-driven inflammatory destruction of the gut epithelial barrier in *Salmonella*-infected mice

**DOI:** 10.1038/s41385-021-00381-y

**Published:** 2021-03-17

**Authors:** Stefan A. Fattinger, Petra Geiser, Pilar Samperio Ventayol, Maria Letizia Di Martino, Markus Furter, Boas Felmy, Erik Bakkeren, Annika Hausmann, Manja Barthel-Scherrer, Ersin Gül, Wolf-Dietrich Hardt, Mikael E. Sellin

**Affiliations:** 1 Institute of Microbiology, Department of Biology, ETH Zurich, Zurich, Switzerland; 2 Science for Life Laboratory, Department of Medical Biochemistry and Microbiology, Uppsala University, Uppsala, Sweden

## Abstract

The gut epithelium is a critical protective barrier. Its NAIP/NLRC4 inflammasome senses infection by Gram-negative bacteria, including *Salmonella* Typhimurium (*S*.Tm) and promotes expulsion of infected enterocytes. During the first ~12–24 h, this reduces mucosal *S*.Tm loads at the price of moderate enteropathy. It remained unknown how this NAIP/NLRC4-dependent tradeoff would develop during subsequent infection stages. In NAIP/NLRC4-deficient mice, *S*.Tm elicited severe enteropathy within 72 h, characterized by elevated mucosal TNF (>20 pg/mg) production from bone marrow-derived cells, reduced regeneration, excessive enterocyte loss, and a collapse of the epithelial barrier. TNF-depleting antibodies prevented this destructive pathology. In hosts proficient for epithelial NAIP/NLRC4, a heterogeneous enterocyte death response with both apoptotic and pyroptotic features kept *S*.Tm loads persistently in check, thereby preventing this dire outcome altogether. Our results demonstrate that immediate and selective removal of infected enterocytes, by locally acting epithelium-autonomous NAIP/NLRC4, is required to avoid a TNF-driven inflammatory hyper-reaction that otherwise destroys the epithelial barrier.

## Introduction

In the mammalian intestine, a continuous single-layered epithelium separates the underlying lamina propria from the dense microbial communities of the lumen, while at the same time providing an extensive surface for fluid and nutrient uptake. Maintenance of this barrier requires tight coordination of mechanisms to remove old and compromised epithelial cells and regenerate replacements from stem cells residing at the bottom of epithelial crypts.^1,2^ Infections and inflammatory responses provide a particular challenge, since both direct injuries by microbes or toxic compounds and pro-inflammatory cytokines, such as tumor necrosis factor (TNF) and interferon gamma (IFNγ) can thwart the delicate epithelial turnover balance.^3–6^ In severe cases, this may lead to the loss of epithelial barrier integrity, which enables direct microbial access to the lamina propria, aggravates tissue pathology, and limits the capacity for fluid and nutrient uptake.^2,7^

Some enteric bacteria can actively invade the gut epithelium. *Salmonella enterica* serovar Typhimurium (*S*.Tm) is a prototype pathogen for studies of invasive gut infection in mice.^8^ *S*.Tm encodes flagella to propel itself towards the mucosa,^9–11^ and a needle-like structure—the type three secretion system 1 (TTSS-1)—to drive invasion of enterocytes.^12–17^ The host has evolved strategies to counteract such insults to the epithelial barrier. This includes the protective effect of the mucus,^9,18,19^ the secretion of antimicrobial peptides,^20^ the recruitment of neutrophils and other inflammatory cell types to eliminate the pathogen,^21–23^ and the production of pathogen-specific IgA that accelerates pathogen clearance from the lumen.^24,25^ In addition, experiments both in vivo and in cultured cells have revealed that inflammasomes, made up of NOD-like receptors (NLRs) and/or inflammatory caspases, are of central importance in the *S*.Tm-infected mucosa.^26^ Several distinct inflammasomes, including canonical NLR/Caspase-1 inflammasomes^27–39^ and non-canonical Caspase-11 (Caspase-4/5 in human) inflammasomes^29,31,40^ have been implicated during *S*.Tm gut infection. Initially, it was assumed that inflammasome responses occur mainly in phagocytes, but it has become evident that also gut epithelial cells use complex inflammasome signaling for antimicrobial defense.^26,41,42^ However, it is still not entirely clear how these inflammasomes are integrated with other mucosal defenses to confer gut protection.

We and others have demonstrated that NAIP1–6 receptors can recognize *S*.Tm virulence factors (i.e., flagellin or TTSS-1),^43–45^ activate a NAIP/NLRC4 inflammasome in vivo,^46,47^ and drive caspase-dependent expulsion of infected enterocytes into the gut lumen.^37,39^ Mice with epithelial-specific ablation of NAIP/NLRC4 show increased *S*.Tm loads in the epithelium, as well as in systemic organs.^39,48^ *S*.Tm promptly downregulates both flagella and TTSS-1 upon epithelial barrier traversal, which explains why epithelial NAIP/NLRC4, but not inflammasomes expressed by professional immune cells, can restrict the initial wave of systemic *S*.Tm spread from the gut lumen during the first day of infection via the oral route.^48^

A functional significance of epithelial NAIP/NLRC4 has now been established,^37,39,48–50^ but several outstanding questions remain. Epithelial NAIP/NLRC4 was reported to act through multiple downstream caspases, i.e., Caspase-1 and/or Caspase-8.^37^ This may in principle result in either lytic or apoptotic cell death, but which of these modalities represents the main mechanism during a physiological infection remains less clear. Partially contrasting conclusions have been drawn from experiments using the hyperstimulus FlaTox vs. live *S*.Tm during in vivo infection.^37,39^ Activation of epithelial NAIP/NLRC4, Caspase-1 or -8 moreover results in release of soluble mediators, including eicosanoids and interleukin-1 family cytokines, most notably interleukin-18 (IL-18).^22,31,32,37^ We found that these cytokines are dispensable for enterocyte expulsion in vivo,^39^ but may accelerate mucosal inflammation in ways that are only beginning to emerge.^51^ These data suggested that NAIP/NLRC4-mediated expulsion of infected epithelial cells may come at the price of a moderate mucosal enteropathy, at least during the first day of infection. Notably, a recent study proposed that IL-18, expressed in a retinoic acid-dependent manner, may also bolster the expulsion of infected enterocytes.^52^ This calls for a systematic revisiting of the cell and tissue wiring of the epithelial NAIP/NLRC4 response. Finally and most importantly, while some studies have shown that NAIP/NLRC4 elicits gut tissue inflammation upon microbial insult,^30,37,39^ others in fact observed exacerbated inflammation and more pronounced tissue destruction in mice lacking NLRC4,^28,49,53^ or an executor Caspase.^33,34,54^ It is unclear whether these seeming contradictions can be explained by analysis within different time-frames of the infection, or rather that they reflect differences between experimental models.

Here, we have assessed the impact and compartmentalization of the mucosal NAIP/NLRC4 defense over time, following oral *S*.Tm infection. We demonstrate that the death and expulsion of infected enterocytes—elicited by local epithelium-autonomous NAIP/NLRC4—is an essential defense to preserve the integrity of the epithelial barrier. In the absence of epithelial NAIP/NLRC4, infected mice develop progressively aggravating mucosal histopathology, abnormally high tissue TNF levels produced by bone marrow (BM)-derived cell type(s), defective epithelial regeneration, and eventually a collapse of the epithelial barrier by 72 h post-infection (p.i.). TNF depletion notably preserves the epithelial barrier integrity of NAIP/NLRC4-deficient animals. These results highlight how epithelium-autonomous NAIP/NLRC4 orchestrates a balanced early response to pathogen invasion, thereby avoiding the tissue-disruptive consequences of an overshooting mucosal immune defense on the epithelial barrier.

## Results

### Epithelial NAIP/NLRC4 preserves epithelium integrity by 72 h of *S*.Tm infection

**Fig. 1:**
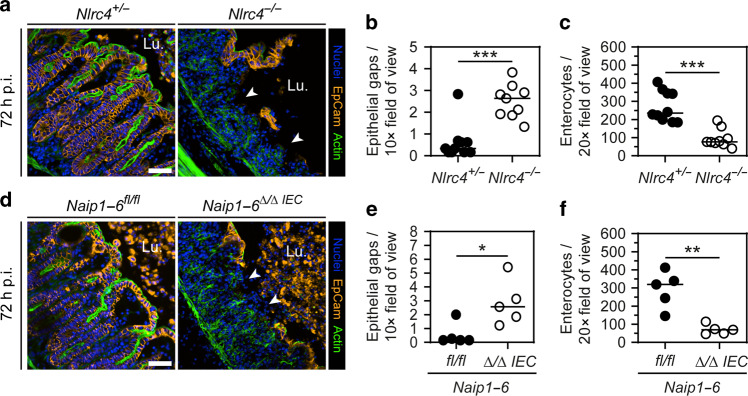
Epithelial integrity loss at 72 h of *S*.Tm infection in NAIP/NLRC4-deficient mice. **a**–**c** NLRC4-deficient mice lose epithelial integrity at late-stage *S*.Tm infection. **a** Representative micrograph of cecal tissue from (streptomycin pretreated) orally *S*.Tm-infected *Nlrc4*^+/−^ control mice and *Nlrc4*^−/−^ littermates at 72 h p.i. White arrowheads indicate epithelial gaps. Lu. lumen. Scale bar: 50 µm. **b** Microscopy-based quantification of epithelial gaps per 10× field of view. Detection limit at 0.17. **c** Microscopy-based quantification of enterocyte numbers within the epithelium per 20× field of view. **d**–**f** Deletion of epithelial NAIP1–6 is sufficient to cause severe epithelial integrity loss. **d** Representative micrograph of cecal tissue from *S*.Tm-infected *Naip1–6*^*fl/*fl^ control mice and *Naip1–6*^*Δ/Δ IEC*^ littermates at 72 h p.i. White arrowheads indicate epithelial gaps. Lu. lumen. Scale bar: 50 µm. **e** Microscopy-based quantification of epithelial gaps per 10× field of view. Detection limit at 0.15. **f** Microscopy-based quantification of enterocyte numbers within the epithelium per 20× field of view. **b**, **c**, **e**, **f** Each data point represents one mouse. Line at median. Five to ten mice per group from ≥2 independent experiments for each comparison. Mann–Whitney *U* test (*p < 0.05, **p < 0.01, ***p < 0.001).

NAIP/NLRC4-dependent expulsion of *S*.Tm-infected enterocytes reduces local and systemic pathogen loads during the first 1–2 days of infection.^37,39,48^ To explore the consequences of this innate NAIP/NLRC4 response on the epithelial barrier during subsequent stages of the infection, we orally infected streptomycin pretreated NLRC4-deficient mice^55^ with 5 × 10^7^ CFUs of wild-type *S*.Tm (SL1344) for 72 h.^56^ In line with previous findings (first 2 days p.i.), we detected elevated *S*.Tm CFUs in mesenteric lymph nodes (mLN), spleen, and liver of *Nlrc4*^−/−^ mice, as compared to heterozygous littermate controls, while luminal colonization appeared equal across genotypes (Fig S1A–D). Strikingly, fluorescence microscopy of cecum (the main site of infection in this model^56^) tissue at 72 h p.i. revealed severe epithelial integrity loss of the *S*.Tm-infected *Nlrc4*^−/−^ mice. The regular villus-crypt architecture was compromised and epithelial fragments (staining positive for the epithelium marker EpCam) were detached, exposing the underlying lamina propria directly to the gut lumen (Fig. 1a). In sharp contrast, heterozygous *Nlrc4*^+/−^ littermate controls still featured an intact continuous epithelium, separating the lamina propria from luminal content (Fig. 1a). In addition, we could observe the typical crypt elongation that is associated with epithelium regeneration in *Nlrc4*^+/−^ control mice, but not in *Nlrc4*^−/−^ littermates (Fig. 1a, Fig S1E). We observed up to 4 epithelial gaps per 10x field of view and significantly reduced total numbers of enterocytes in the mucosal tissue of NLRC4-deficient mice (Fig. 1b, c). Hence, NLRC4 is required to prevent loss of intestinal epithelium integrity by 72 h of acute *S*.Tm infection. These results appeared somewhat surprising, since our previous work in fact demonstrated that NAIP/NLRC4 actively promotes enterocyte expulsion during the first day of infection.^39^

Previous work has shown that NAIP/NLRC4 specifically within epithelial cells counteracts acute *S*.Tm infection.^37,39,48^ Hence, we sought to address whether preservation of gut epithelium integrity at 72 h could also be assigned to epithelial NAIP/NLRC4. We infected epithelial-specific NAIP1–6 knockout mice (*Naip1–6*^*Δ/ΔIEC*^;^57^) for 72 h. Similar to *Nlrc4*^−/−^ mice, and in line with previous data,^48^ *Naip1–6*^*Δ/ΔIEC*^ mice showed normal luminal colonization, but increased systemic *S*.Tm loads (Fig S1F–I). Importantly, the specific deletion of epithelial NAIPs was sufficient to cause severe epithelium integrity loss at 72 h p.i. (Fig. 1d). We again observed increased numbers of epithelial gaps and reduced total enterocyte numbers in the mucosal tissue of *Naip1–6*^*Δ/ΔIEC*^ animals compared to the controls, mimicking the effect of full-body NLRC4-ablation (Fig. 1e, f, compare with 1b–c). Taken together, these observations suggest that epithelial NAIP/NLRC4 prevents gut epithelium integrity loss by 72 h post *S*.Tm infection.

### Prompt NAIP/NLRC4-driven enterocyte expulsion through a heterogeneous cell death response commences by 9 h of *S*.Tm infection

In our earlier work, mice deficient in NAIP/NLRC4 were shown to expel fewer *S*.Tm-infected enterocytes into the gut lumen, at least during the first 12–18 h of infection.^39^ Therefore, it appeared somewhat counterintuitive that here we observed epithelium loss in NAIP/NLRC4-deficient mice at 72 h p.i.. To disentangle how epithelial NAIP/NLRC4 prevents epithelium integrity loss at 72 h of *S*.Tm infection, we studied the impact of NLRC4-ablation over the entire 72 h. Prior to infection, enterocyte cell death and epithelial turnover rates were similar between *Nlrc4*^−/−^ mice and heterozygous littermate controls (Fig S2A–F). Furthermore, we could not detect any significant differences in steady state inflammation parameters (Fig S2G–K).

**Fig. 2:**
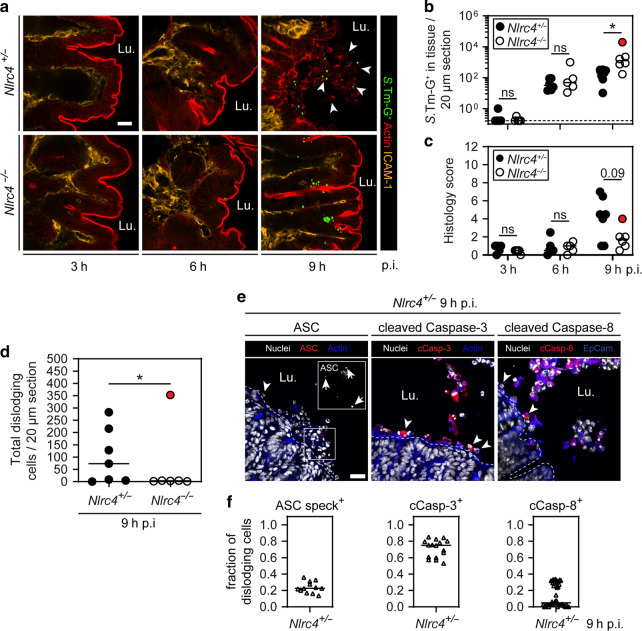
NAIP/NLRC4 provides a first line defense by promoting the prompt expulsion of *S*.Tm-infected enterocytes. **a**–**c** Early *S*.Tm infection kinetics of NLRC4-sufficient and deficient mice. **a** Representative micrographs of cecal tissue from *S*.Tm/*pssaG-GFP*-infected *Nlrc4*^+/−^ control mice and *Nlrc4*^−/−^ littermates at 3, 6, and 9 h p.i. White arrowheads indicate dislodged enterocytes. Lu. lumen. Scale bar: 20 µm. **b** Microscopy-based quantification of intracellular *S*.Tm-G^+^ in cecal epithelium per 20 µm cross-section at 3, 6, and 9 h p.i. Dashed line represents detection limit. **c** Histology score of cecal tissue at 3, 6, and 9 h p.i. **d** Microscopy-based quantification of dislodging enterocytes per 20 µm cross-section at 9 h p.i. Detection limit at 0.25. **b**–**d** Each data point represents one mouse. Line at median. Data points marked in red belong to one single NLRC4-deficient mouse with unusually progressed infection. This mouse was excluded in the statistical analysis. Five to seven mice per group from ≥2 independent experiments for each comparison. **e**, **f** Microscopy-based characterization of NLRC4-dependent enterocyte expulsion. **e** Representative micrographs of cecal tissue from *Nlrc4*^+/−^ mice at 9 h p.i. stained for ASC, cleaved Caspase-3 and cleaved Caspase-8. White arrowheads indicate dislodging cells. White arrows indicate ASC specks. Lu. lumen. Scale bar: 20 µm. **f** Microscopy-based quantification of the indicated markers in dislodging enterocytes. Each data point represents one field of view. Tissue sections from three to four mice quantified. **b**–**d** Mann–Whitney *U* test (ns—not significant, *p < 0.05).

To survey the kinetics of early infection, *Nlrc4*^−/−^ mice and heterozygous littermate controls were infected as above with *S*.Tm harboring a p*ssaG-GFP* reporter (renders *S*.Tm GFP-positive upon host cell entry; hereafter denoted *S*.Tm-G^+^,^15^). Already at 3 h p.i. the cecal lumen contained substantial numbers of *S*.Tm, and within 6 h was fully colonized, reaching densities of ~10^9^ CFUs per gram (Fig S3A, mLN counts shown in Fig S3B). At 6 h p.i., we also observed the first *S*.Tm within the epithelium (Fig. 2a, b). This extends our previous work by demonstrating that the *S*.Tm invasion efficiency into enterocytes is not affected by NLRC4, since initial epithelial *S*.Tm loads were similar between the two backgrounds (Fig. 2b, compare *Nlrc4*^+/−^ and *Nlrc4*^−/−^ at 6 h p.i.). At 9 h p.i., however, we observed significantly more intra-epithelial *S*.Tm and a trend towards the delayed onset of inflammation in NLRC4-deficient mice than in NLRC4-proficient littermates (Fig. 2b, c). Anti-*S*.Tm-LPS staining revealed a similar increase in intra-epithelial bacteria, hence excluding any reporter-dependent bias (Fig S3C, D). At this time point, control animals showed pronounced expulsion of infected enterocytes, while we could not detect dislodging enterocytes in 5 out of 6 *Nlrc4*^−/−^ mice (Fig. 2a, d). Notably, due to inter-individual variation, one single *Nlrc4*^−/−^ mouse already harbored exceptionally high epithelial *S*.Tm loads (>10^4^/tissue section) and showed signs of inflammation (red dot in Fig. 2b, c), typically observed at later stages of the infection (~18 h p.i.) in this genetic background.^39^ In this one animal, dislodged epithelial cells were also frequently found in the lumen (Fig. 2d, red dot). These findings indicate that NLRC4-dependent enterocyte expulsion is a prompt first line response to reduce epithelial *S*.Tm loads and elicit inflammation, but that NLRC4-independent epithelial cell dislodging can also occur, as the infection progresses.

Next, we addressed the features of the prompt NLRC4-dependent enterocyte expulsion response at 9 h p.i.. To this end, cecal sections from the heterozygous control group were stained for inflammasome and cell death markers. We chose cecal samples from 9 h p.i., where enterocyte expulsion had begun and influx of blood-derived cell types into the lumen was negligible (Fig S3E–F). Some, but not all, dislodging enterocytes harbored specks of the inflammasome adaptor protein ASC (~20%), indicating classical inflammasome activation (Fig. 2e, f). ASC specks are partly dispensable for NLRC4 downstream signaling,^55,58,59^ which could explain the large fraction of dislodging enterocytes without ASC specks. The majority of the dislodging enterocytes were positive for cleaved Caspase-3 (~80%, Fig. 2e, f). By contrast, cleaved Caspase-8 staining was highly variable. In some mice, up to 30% of dislodging enterocytes were positive for cleaved Caspase-8, while in others, cleaved Caspase-8 staining was undetectable. This extends the findings of a previous report, which observed multiple potential cell death cascades downstream of NLRC4 upon FlaTox challenge.^37^ In summary, these results establish that (1) epithelial NAIP/NLRC4 drives a swift and heterogeneous enterocyte death response to reduce *S*.Tm tissue loads in the very first window of acute infection, but also that (2) NAIP/NLRC4-independent enterocyte dislodging could occur at subsequent stages of the infection.

### Locally acting epithelium-autonomous NAIP/NLRC4 elicits the early death and expulsion of infected enterocytes with mixed apoptotic and pyroptotic features

NAIP/NLRC4 drives expulsion of infected enterocytes (Fig. 2;^37,39^), and hyper-activation with the pure NAIP5-6 ligand FlaTox can elicit enterocyte death, with cell lysis preceding expulsion through a process similar to macrophage pyroptosis.^37^ However, it remained to be tested if these conclusions hold true for the NAIP/NLRC4-dependent epithelial response to live *S*.Tm. To answer these questions, we established intestinal epithelial organoids (pure epithelium cultures, hereafter denoted “enteroids”;^60,61^) from the indicated genetic mouse backgrounds, and infected these in bulk with *S*.Tm/p*ssaG-GFP*. We detected significantly more *S*.Tm-G^+^ infection foci, and higher total *S*.Tm CFUs in 3D enteroids derived from *Nlrc4*^−/−^ mice than in WT control enteroids at ~4 h p.i. onwards (Fig S4A–C). Similar results were obtained in internally controlled mixed 3D cultures of WT (non-colored) and NAIP1–6-deficient (red) enteroids (from *RFP*^*IEC*^*Naip1–6*^*Δ/ΔIEC*^ mice; see Fig S4D for in vivo validation) (Fig S4E, F). Mixed cultures of RFP-expressing WT and non-colored *Nlrc4*^−/−^ enteroids also featured higher levels of *S*.Tm-G^+^ in the *Nlrc4*^−/−^ subpopulation (Fig S4G, H).

**Fig. 3:**
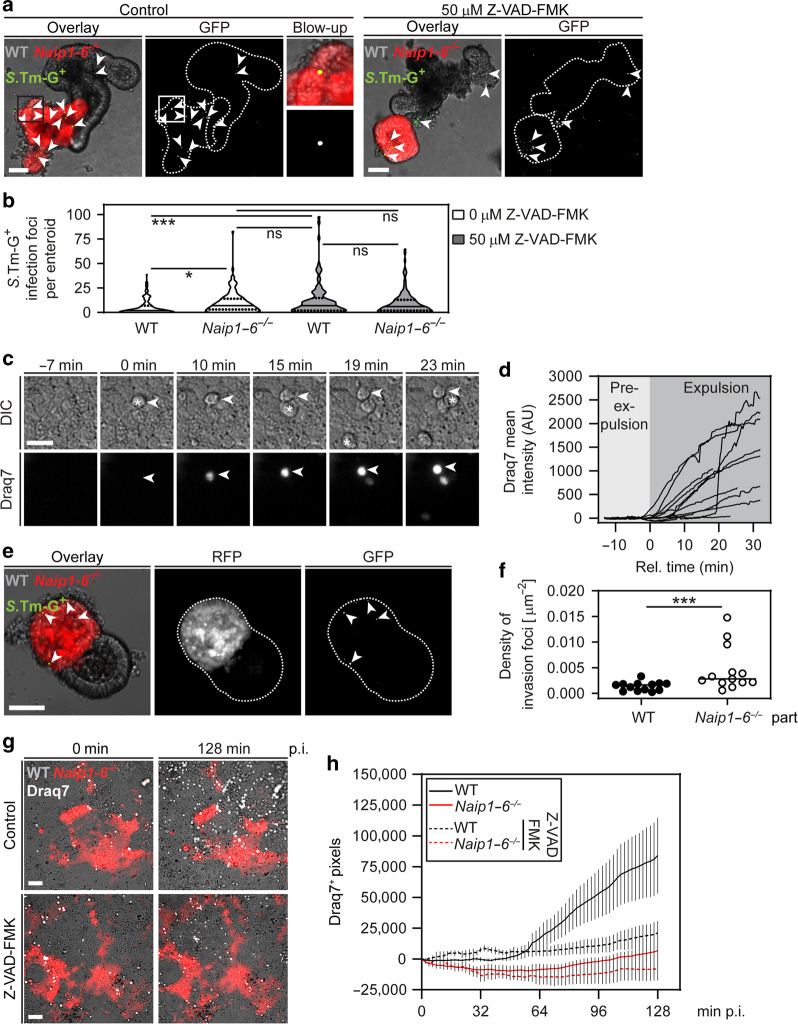
Locally acting epithelium-autonomous NAIP/NLRC4 drives caspase-dependent expulsion of enterocytes with an initially Draq7-impermeable membrane. **a**, **b** Pan-caspase inhibition phenocopies NAIP1–6 deficiency with respect to epithelial *S*.Tm loads in mixed enteroid cultures. **a** Representative micrographs of *S*.Tm/*pssaG-GFP*-infected (MOI 100) WT and RFP-expressing NAIP1–6-deficient enteroids in the absence or presence of 50 µM pan-caspase inhibitor Z-VAD-FMK at 4 h p.i. Arrowheads indicate intracellular *S*.Tm-G^+^ infection foci. Scale bar: 50 µm. **b** Microscopy-based quantification of intracellular *S*.Tm-G^+^ infection foci per enteroid. In violin plot, line represents median and dashed line quartiles. **c**, **d** Cell lysis during early enterocyte expulsion. **c** Representative time-lapse microscopy image series of *S*.Tm-infected enteroid-derived 2D monolayers (MOI 2). Cell lysis was determined using the membrane impermeable nuclear dye Draq7. Example of an expelling enterocyte indicated by arrowhead - time point 0 represents the first sign of expulsion, as judged from differential interference contrast (DIC) microscopy. Star represents first sign of expulsion. Scale bar: 20 µm. **d** Quantification of Draq7 mean intensity of individual enterocytes during expulsion. Time point 0 represents the first signs of expulsion, as judged from DIC microscopy. **e**, **f** Chimeric 3D enteroids show higher *S*.Tm loads in NAIP1–6-deficient epithelial regions. **e** Representative micrographs of an *S*.Tm/*pssaG-GFP*-infected WTx*Naip1–6*^−/−^ (red; *RFP*^*IEC*^*Naip1–6*^*Δ/ΔIEC*^) chimeric 3D enteroid (MOI 100) at 4 h p.i. Arrowheads indicate intracellular *S*.Tm-G^+^ infection foci. Scale bar: 50 µm. **f** Microscopy-based quantification of intracellular *S*.Tm-G^+^ infection foci density. Line at median. Each data point represents one enteroid. **g**, **h** Enterocyte death in WTx*Naip1–6*^−/−^ (red; *RFP*^*IEC*^*Naip1–6*^*Δ/ΔIEC*^) chimeric enteroid-derived 2D monolayers. **g** Representative micrographs of *S*.Tm-infected WTx*Naip1–6*^−/−^ chimeric monolayers at MOI 2 over time in the absence or presence of 50 µM Z-VAD-FMK. Draq7 was added to the medium to visualize enterocyte cell death (end stage, see **c** above). Scale bar: 50 µm. **h** Quantification of Draq7^+^ pixel numbers relative to the start of the imaging series. Results in **a**–**d** and **g**, **h** representative for three independent infections. **e**, **f** Present data from one infection experiment (see also Fig S6B for an additional supporting experiment). **b** Two-way ANOVA with Tukey HSD and **f** Mann–Whitney *U* test (ns—not significant, *p < 0.05, ***p < 0.001).

To probe the significance of Caspase signaling, mixed cultures of WT (non-colored) and *RFP*^*IEC*^*Naip1–6*^*Δ/ΔIEC*^ (red) enteroids were infected in the presence or absence of the pan-caspase inhibitor Z-VAD-FMK (50 µM; effect of different concentrations was probed in WT enteroids; Fig S5A). In line with results above, untreated *RFP*^*IEC*^*Naip1–6*^*Δ/ΔIEC*^ enteroids accumulated higher numbers of *S*.Tm-G^+^ infection foci than WT counterparts (Fig. 3a, b). Treatment with Z-VAD-FMK, however, nullified this difference (Fig. 3a, b). These results extend previous studies,^29,37,39^ and unequivocally show that epithelium-autonomous NAIP/NLRC4 drives Caspase-dependent restriction of *S*.Tm gut epithelium infection.

Previous work by different methodologies had yielded conflicting results with respect to the relative timing of NAIP/NLRC4-dependent expulsion and plasma membrane permeabilization.^37,39^ Therefore, we re-examined this question in enteroid-derived 2D monolayer cultures, infected from the apical side. Upon *S*.Tm infection, we noted prompt expulsion of enterocytes from the monolayer (Fig. 3c, Fig S5B). Infections with *S*.Tm-mCherry confirmed that expelling enterocytes harbored bacteria (Fig S5C). In support of the heterogeneous cell death response observed in vivo (Fig. 2e), expelling enterocytes lost plasma membrane integrity at different stages of expulsion, as judged by uptake of the DNA-binding dye Draq7 added to the medium (Fig. 3c, d). Importantly, most enterocytes showed clear signs of expulsion (determined by differential interference contrast (DIC) microscopy) before Draq7 uptake began, indicating that initiation of the expulsion process precedes overt membrane integrity loss (Fig. 3c, d). Prior to expulsion, some infected enterocytes within the monolayer stained positive for cleaved Caspase-3 (6.5 ± 3.9% of all infected cells), but not for Draq7^+^ (Fig S5D, E). Nevertheless, most expelling enterocytes eventually became Draq7^+^ at later stages of the time series.

Epithelium-autonomous NAIP/NLRC4 could in principle act either locally at the site of the infected cell, or alternatively extend across the entire connected epithelium. To discriminate between these possibilities, we generated 3D chimeric enteroids comprised of genetically distinct epithelial regions. First, WT and RFP-expressing enteroids were seeded densely together after harsh fragmentation. At 2 days post embedding, we indeed found a subpopulation of chimeric enteroids (~1% of all), comprised of alternating red and non-colored regions (Fig S6A). The same approach was used to create chimeras from non-colored WT and red *RFP*^*IEC*^*Naip1–6*^*Δ/ΔIEC*^ enteroid fragments. When infected, *RFP*^*IEC*^*Naip1–6*^*Δ/ΔIEC*^ regions accumulated significantly more *S*.Tm-G^+^ infection foci compared to WT regions (Fig. 3e, f; presented as foci per area to enhance the data precision). A similar trend was observed in chimeras composed of RFP-expressing WT and non-colored *Nlrc4*^−/−^ regions (Fig S6B, C). This hinted towards a local epithelial NAIP/NLRC4 circuit restricting *S*.Tm infection, but the low efficiency of 3D chimera generation hampered deeper analysis. Using a similar principle, we established chimeric 2D monolayers from WT and *RFP*^*IEC*^*Naip1–6*^*Δ/ΔIEC*^ enteroids, and followed *S*.Tm infection (MOI 2) over ~3 h by time-lapse microscopy. Draq7 was again added to the culture media to label membrane-permeabilized (dead) enterocytes. This analysis revealed a gradual accumulation of expelled/dying enterocytes within WT, but not NAIP1–6-deficient, regions of the monolayer (Fig. 3g, h). Importantly, this difference disappeared in the presence of Z-VAD-FMK treatment, which specifically reduced enterocyte cell death in WT regions back to baseline (Fig. 3g, h).

Taken together with our in vivo observations (Fig. 2), these results demonstrate that local epithelium-autonomous NAIP/NLRC4 induces a prompt, heterogeneous, Caspase-dependent cell death response, which fuels expulsion of *S*.Tm-infected enterocytes that gradually lose their plasma membrane integrity. However, these data alone were insufficient to explain why NAIP/NLRC4-deficient mice suffer from such massive epithelial disruption after 72 h of *S*.Tm infection (Fig. 1).

### Enterocyte dislodgement can occur independently of NAIP/NLRC4 and involves TNF signaling

**Fig. 4:**
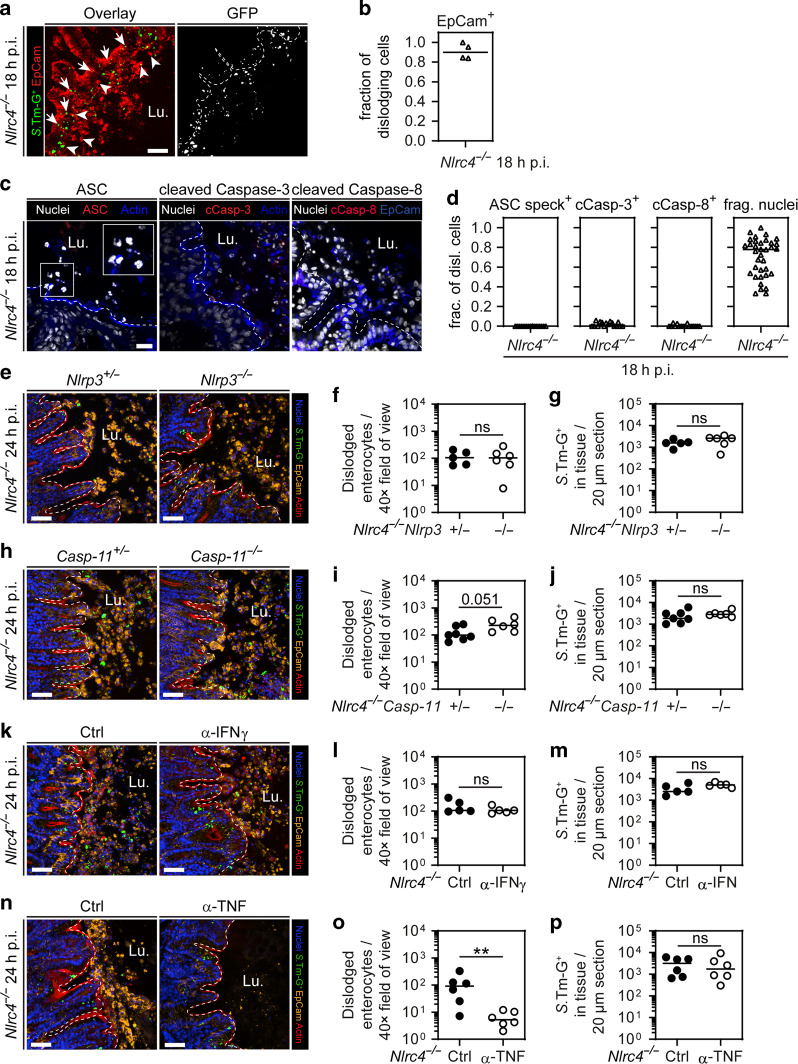
Pronounced NLRC4-independent dislodging of enterocytes at 24 h p.i. involves TNF. **a**, **b** NLRC4 deficiency leads to pronounced epithelial erosion. **a** Representative micrograph of cecal tissue from *S*.Tm/*pssaG-GFP*-infected *Nlrc4*^−/−^ mice at 18 h p.i. This pronounced erosion can only be seen occasionally in a few mice at this time point. Arrows indicate epithelial *S*.Tm-G^+^. Arrowheads indicate dislodged enterocytes with and without intracellular *S*.Tm-G^+^. Lu. lumen. Scale bar: 50 µm. **b** Microscopy-based quantification of enterocyte fraction among dislodging cells in **a**. Line at median. **c**, **d** Microscopy-based characterization of dislodging enterocytes. **c** Representative micrographs of cecal tissue from *Nlrc4*^−/−^ mice at 18 h p.i. stained for ASC, cleaved Caspase-3 and cleaved Caspase-8. Lu. lumen. Scale bar: 20 µm. **d** Microscopy-based quantification of the indicated markers and fragmented nuclei in dislodging enterocytes. Each data point represents one field of view. Four mice quantified. Line at median. **e**–**g** *S*.Tm/*pssaG-GFP*-infection of *Nlrc4*^−/−^*Nlrp3*^+/−^ control mice and *Nlrc4*^−/−^*Nlrp3*^−/−^ littermates for 24 h. **e** Representative micrographs of cecal tissue. **F** Microscopy-based quantification of dislodged enterocytes per 40× field of view. **g** Microscopy-based quantification of intracellular *S*.Tm-G^+^ in cecal epithelium. **h**–**j** *S*.Tm/*pssaG-GFP* infection of *Nlrc4*^−/−^*Caspase11*^*+/−*^ control mice and *Nlrc4*^−/−^*Caspase11*^−/−^ littermates for 24 h. **h** Representative micrographs of cecal tissue. **I** Microscopy-based quantification of dislodged enterocytes per 40× field of view. **j** Microscopy-based quantification of intracellular *S*.Tm-G^+^ in cecal epithelium. **k**–**m** *S*.Tm/*pssaG-GFP* infection of *Nlrc4*^−/−^ mice for 24 h, injected (i.p.) with anti-IFNγ or isotype control antibody (twice daily dose of 500 µg antibodies per mouse, starting 1 day before infection^103^). **k** Representative micrographs of cecal tissue. **l** Microscopy-based quantification of dislodged enterocytes per 40× field of view. **m** Microscopy-based quantification of intracellular *S*.Tm-G^+^ in cecal epithelium. **n**–**p** *S*.Tm/*pssaG-GFP* infection of *Nlrc4*^−/−^ mice for 24 h, injected (i.p.) with anti-TNF or isotype control antibody (once with 500 µg antibodies per mouse 1 day before infection). **n** Representative micrographs of cecal tissue. **o** Microscopy-based quantification of dislodged enterocytes per 40× field of view. **p** Microscopy-based quantification of intracellular *S*.Tm-G^+^ in cecal epithelium. **e**, **h**, **k**, **n** Lu.—lumen, scale bars: 50 µm. **f**, **g**, **i**, **j**, **l**, **m**, **o**, **p** Each data point represents one mouse. Line at median. Five to seven mice per group from ≥2 independent experiments for each comparison. Note: Expression and maturation of GFP reporter takes around 2–4 h. Mann–Whitney *U* test (ns—not significant, **p < 0.01).

In the earlier in vivo experiments, we observed one NLRC4-deficient mouse with exceptionally high epithelial *S*.Tm loads, featuring signs of inflammation and high numbers of dislodging enterocytes at 9 h p.i. (red dot in Fig. 2b–d). Therefore, we hypothesized that high tissue *S*.Tm loads may eventually induce NAIP/NLRC4-independent mechanism(s) for enterocyte dislodgment and inflammation. To address this, we first extended the in vivo infection kinetics experiments to 18 h p.i and focused on dislodging enterocytes in *Nlrc4*^−/−^ mice. Notably, in some NLRC4-deficient mice, we could detect *S*.Tm invasion hot spots, where high numbers of EpCam-positive enterocytes simultaneously dislodged from the epithelium (Fig. 4a, b). These luminal enterocytes displayed a morphologically compromised cell shape and some, but far from all, contained *S*.Tm-G^+^ infection foci (Fig. 4a). In sharp contrast to the early NAIP/NLRC4-induced enterocyte expulsion (Fig. 2e, f), ASC speck formation as well as distinct activation of Caspase-3 and Caspase-8 was not observed at this time point (Fig. 4c, d, compare to Fig. 2e, f). We could only detect a faint cleaved Caspase-3 staining, which showed marginal increase in the fluorescence intensity compared to secondary antibody control sections (Fig S7A). These data suggest that accumulation of *S*.Tm in the gut epithelium eventually causes dislodgement of enterocytes also in the absence of NAIP/NLRC4, through a process qualitatively distinct from the initial NAIP/NLRC4-dependent enterocyte expulsion. Still, analysis of nuclear morphology among dislodging enterocytes hinted towards that also this response may be variable in nature (Fig. 4d; rightmost panel).

Autophagy, other inflammasomes, such as NLRP3 and Caspase-11, and pro-inflammatory cytokines, such as TNF and IFNγ, have all been implicated in *S*.Tm infection.^27,29,31,62–69^ However, in littermate-controlled infections, we found that these factors/pathways were dispensable for restriction of epithelial *S*.Tm loads in the presence of functional NAIP/NLRC4 (i.e., in a WT mouse background) at 18 h p.i. (Fig S7B–E, and refs. ^39,68^). We next explored the effects that such host defenses may have in the absence of NAIP/NLRC4. Specifically, we hypothesized that pathways previously linked to cell death induction in different contexts—e.g., NLRP3,^70^ Caspase-11,^71^ IFNγ,^72^ or TNF^73,74^—might induce the enterocyte dislodging we observed in NAIP/NLRC4-deficient animals at ~18 h p.i. onwards.

*Nlrc4*^−/−^ mice were interbred with *Nlrp3*^−/−^ or *Caspase-11*^−/−^ animals, and back-crossed to generate either *Nlrc4*^−/−^*Nlrp3*^−/−^ or *Nlrc4*^−/−^*Caspase-11*^−/−^ double knockout mice and their corresponding *Nlrc4*^−/−^*Nlrp3*^+/−^ or *Nlrc4*^−/−^*Caspase-11*^+/−^ littermate controls. These animals were infected for 24 h, resulting in equal luminal colonization across genotypes (Fig S7F and S7I). Moreover, we observed similar numbers of dislodged enterocytes between *Nlrc4*^−/−^*Nlrp3*^−/−^ or *Nlrc4*^−/−^*Caspase-11*^−/−^ double knockouts and their respective heterozygous littermates (Fig. 4e, f, h, i). If anything, there seemed to be a slight trend towards higher numbers of dislodging enterocytes in *Nlrc4*^−/−^*Caspase-11*^−/−^ mice. Epithelial *S*.Tm loads were also similar across the genotypes, based on both microscopy and CFU plating assays (Fig. 4g, j, S7G, J). Finally, no differences in histology scores were observed (Fig S7H, K). These findings imply a negligible contribution of NLRP3 and/or Caspase-11 to the enterocyte dislodging phenomenon seen in *Nlrc4*^−/−^ animals at 24 h p.i.

Next, we addressed the effect of the potential cell death-inducing cytokines IFNγ and TNF. In vivo antibody depletion of IFNγ in NLRC4-deficient mice did not influence the numbers of dislodging enterocytes and the overall histology score, nor did it have any effect on epithelial *S*.Tm loads at 24 h p.i. (Fig. 4k–m, Fig S7L–N). By sharp contrast, TNF depletion reduced the numbers of dislodging enterocytes by up to ~20-fold in *Nlrc4*^−/−^ mice at this time point (Fig. 4n–o, Fig S7O–Q). Notably however, we could not detect higher epithelial *S*.Tm loads upon anti-TNF treatment (Fig. 4p, Fig S7P). This might be explained by the fact that TNF causes indiscriminate dislodging of enterocytes,^75^ rather than the selective expulsion of infected enterocytes elicited early by NAIP/NLRC4-proficient hosts (Figs. 2–3 and ref. ^39^). In summary, abundant enterocyte dislodging occurs in NAIP/NLRC4-deficient animals at mature stages of *S*.Tm infection, through a mechanism that involves TNF signaling.

### NAIP/NLRC4 deficiency leads to elevated TNF levels, erosion of the epithelial barrier, and compromised tissue regeneration by 24–72 h of *S*.Tm infection

**Fig. 5:**
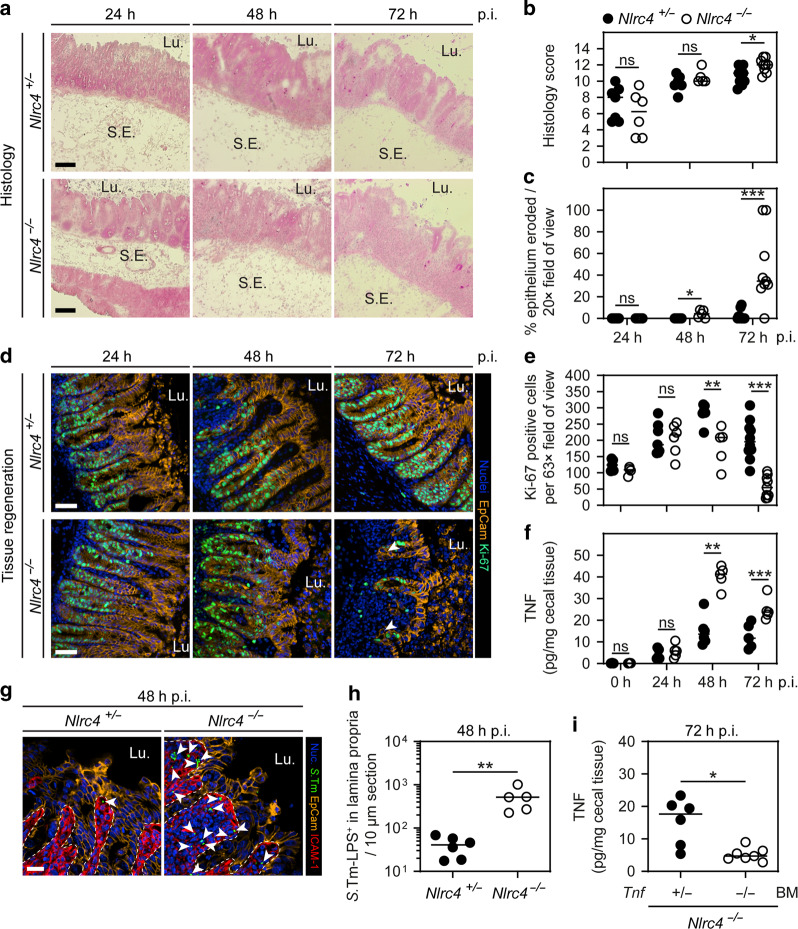
Kinetics of mucosal pathology in *S*.Tm-infected NLRC4-deficient mice at 24–72 h p.i. **a**–**c** Cecal histology of littermate controls and NLRC4-deficient mice over 3 days of *S*.Tm infection. **a** Representative micrographs of cryo-embedded H&E-stained cecal tissue sections at 24, 48, and 72 h p.i. Lu. lumen, S.E. submucosa edema. Scale bars: 100 µm. **b** Microscopy-based quantification of histology score. **c** Microscopy-based quantification of percentage epithelium eroded. H&E-stained tissue sections as in **a** were used for quantification. Detection limit at 0.3%. **d**, **e** Epithelial cell proliferation of littermate controls and NLRC4-deficient mice over 3 days of *S*.Tm infection. **d** Representative micrographs of cecal tissue sections, stained for cell proliferation marker Ki-67 at 24, 48, and 72 h p.i. Arrowheads indicate crypt cells without Ki-67 staining. Lu. lumen. Scale bars: 50 µm. **e** Microscopy-based quantification of Ki-67 positive epithelial cells per 63× field of view. **d**, **e** Specific areas without epithelial gaps were chosen. **f** TNF concentrations in cecal tissue at 0, 24, 48, and 72 h p.i. **g**, **h** Lamina propria *S*.Tm in NLRC4-deficient and littermate control mice at 48 h p.i. **g** Representative micrographs of cecal tissue sections, stained for *S*.Tm-LPS at 48 h p.i. Arrowheads indicate *S*.Tm in lamina propria. Lu. lumen, scale bar: 20 µm. **h** Microscopy-based quantification of *S*.Tm-LPS^+^ cells in the lamina propria. **i** TNF concentrations in cecal tissue of bone marrow chimeras at 72 h p.i. BM bone marrow. **b**, **c**, **e**, **f**, **h**, **i** Each data point represents one mouse. Line at median. Five to ten mice per group from ≥2 independent experiments per comparison (except 0 h time point in **f**, three mice per group). Mann–Whitney *U* test (ns—not significant, *p < 0.05, **p < 0.01, ***p < 0.001).

To trace the onwards progression of the infection in the presence or absence of NAIP/NLRC4, we extended our kinetic experiments to the 24–72 h p.i. window. Since the expression of GFP attenuates *S*.Tm fitness over time, which becomes apparent after >24 h p.i., we infected *Nlrc4*^+/−^ and *Nlrc4*^−/−^ littermates with *S*.Tm lacking the p*ssaG-GFP* reporter. Gut luminal *S*.Tm densities were similar across genotypes throughout the infection (Fig S8A). Hematoxylin and Eosin staining of cecal tissue revealed a progressing enteropathy in both the control and the NLRC4-deficient mice (Fig. 5a, b). Epithelial barrier integrity remained largely intact up to 48 h p.i. in both genotypes (Fig. 5c). Thereafter, however, an abrupt collapse of the epithelial layer was observed specifically in the NLRC4-deficient animals (Fig. 5a, c, 72 h p.i.; supported by the results in Fig. 1a–f). Dislodging enterocytes at this stage featured a variety of cell death markers and the overall tissue architecture was disrupted (Fig S8B).

Simultaneously to the loss of epithelial integrity, we observed an irregular villus-crypt architecture in *Nlrc4*^−/−^ mice at 72 h p.i. (Figs. 1a–c and 5a, b). This might be the consequence of a compromised stem cell niche, which normally increases proliferation to compensate for the loss of epithelial cells during conditions of excessive cell death. In line with a recent study claiming stem cell loss during DSS-induced inflammation,^76^ we observed reduced total mRNA levels of the stem cell markers Lgr5 and Ascl2 in cecal tissue of NLRC4-deficient mice at 48–72 h p.i. (Fig S8C, D). We next stained cecal tissue sections for the cell proliferation marker Ki-67.^77^ At 24 h p.i., we quantified increased crypt cell proliferation compared to steady state in both *Nlrc4*^+/−^ and *Nlrc4*^−/−^ mice. This indicates that loss of enterocytes both by NAIP/NLRC4-dependent expulsion and by NAIP/NLRC4-independent mechanism(s) can initially be compensated through enhanced crypt cell proliferation (Fig. 5d, e). However, while the numbers of proliferative cells further increased in heterozygous control mice at 48 h p.i., NLRC4-deficient mice reached a plateau and had essentially lost their proliferative compartment by 72 h p.i. (Fig. 5d, e). Hence, NAIP/NLRC4 is critical for preservation of both the differentiated and the proliferative regions of the gut epithelial barrier during progressed *S*.Tm infection.

Our earlier data suggested an involvement of TNF (Fig. 4n–o), which is known to have pleiotropic effects. On the one hand, TNF-induced NFκB signaling is associated with cell survival, proliferation, cytokine production and inflammation, while on the other hand, TNF can induce Caspase-dependent and -independent cell death responses.^73,74^ To assess the role of TNF in more detail, we measured TNF protein concentrations in the cecal tissue of *S*.Tm-infected *Nlrc4*^+/−^ and *Nlrc4*^−/−^ mice over time. At 24 h p.i., we observed roughly similar intermediate TNF concentrations (~3–6 pg/mg) across the genotypes in response to *S*.Tm infection (Fig. 5f). Strikingly, at 48 h p.i. TNF concentrations spiked to ~40 pg/mg (>7-fold increase) in *Nlrc4*^−/−^ mice, while *Nlrc4*^+/−^ controls only showed a modest increase (Fig. 5f). This difference persisted also at 72 h p.i. (Fig. 5f).

We next aimed to identify the source of elevated TNF in this context. Notably, during the critical transition at ~48 h p.i., we found abnormally high *S*.Tm loads (~10–20-fold over controls) specifically in the lamina propria compartment of *Nlrc4*^−/−^ mice (Fig. 5g, h; epithelial loads in Fig. S8E). This suggested that in the absence of the sensitive NAIP/NLRC4 defense, immune cells in the lamina propria might experience hyperstimulation by *Salmonella* ligands, resulting in excessive TNF production. To test this hypothesis, *Nlrc4*^−/−^ mice were gamma-irradiated, and the BM reconstituted from either *Tnf*^+/−^ or *Tnf*^−/−^ donors. When infected with *S*.Tm, recipient mice that received *Tnf*^+/−^ or *Tnf*^−/−^ BM exhibited similar luminal S.Tm colonization (Fig S8F). However, only *Tnf*^+/−^ BM recipients produced high levels of TNF (Fig. 5i). Furthermore, in the *Tnf*^+/−^ BM recipients where TNF levels were the highest (≥20 pg/mg), disruption of the epithelial barrier was again seen (Fig S8G–I).

Taken together, our findings suggest that NAIP/NLRC4 deficiency leads to epithelial barrier erosion, compromised tissue regeneration, and excessive production of TNF by BM-derived mucosal cell types during late-stage *S*.Tm infection. However, it remained to be formally shown if the increase in mucosal TNF from ~3–6 to ~40 pg/mg would suffice to explain the collapse of the epithelial barrier.

### Exacerbated TNF signaling in NLRC4-deficient mice promotes the demise of the epithelial barrier at 72 h p.i

**Fig. 6:**
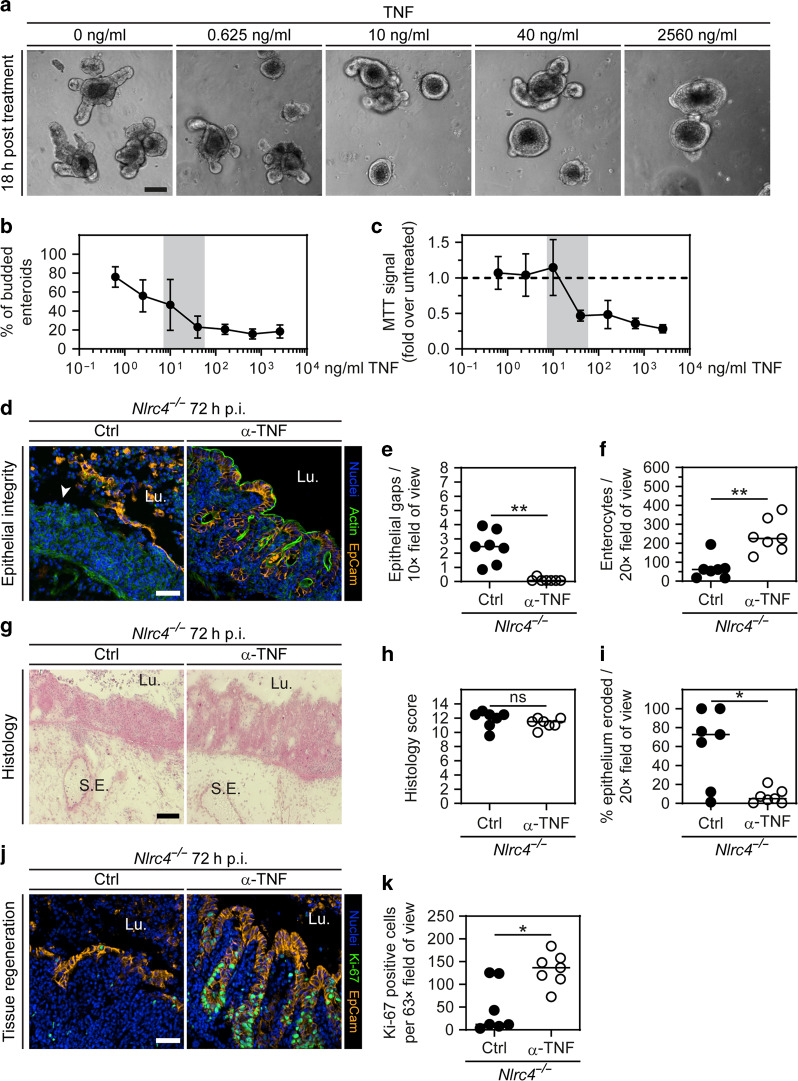
TNF depletion preserves epithelial barrier integrity in NLRC4-deficient mice at 72 h p.i. **a**–**c** Effect of TNF on enteroids. **a**, **b** Enteroids were exposed to TNF 3 days post seeding. **a** Representative micrographs of enteroids at 18 h of treatment with different concentrations of TNF. Scale bar: 100 µm. **b** Microscopy-based quantification of percentage budded enteroids. **c** Enteroids were exposed to TNF directly after seeding. MTT assay at 6 days post seeding. Fold change of light absorption at 562 nm over untreated is plotted. **b**, **c** Each data point represents the mean with SD of ≥4 separately treated cultures. Gray area represents the approximate range of TNF concentration measured in littermate controls and *Nlrc4*^−/−^ mice (derived from the data in Fig. 5f). **d**–**k** 72 h *S*.Tm infection of *Nlrc4*^−/−^ mice treated with isotype control or anti-TNF. **d**–**i** TNF depletion preserves epithelial integrity in NLRC4-deficient mice. **d** Representative micrographs of cecal tissue from littermate controls and *Nlrc4*^−/−^ mice at 72 h p.i. White arrowhead indicates epithelial gap. Lu. lumen. Scale bar: 50 µm. **e** Microscopy-based quantification of epithelial gaps per 10× field of view. Detection limit at 0.07. **f** Microscopy-based quantification of enterocytes per 20× field of view. **g** Representative micrographs of cryo-embedded H&E-stained cecal tissue sections at 72 h p.i. Lu. lumen, S.E. submucosa edema. Scale bar: 100 µm. **h** Microscopy-based quantification of histology score. **i** Microscopy-based quantification of percentage epithelium eroded. H&E-stained tissue sections as in **g** were used for quantification. Detection limit at 0.6%. **j**, **k** TNF depletion preserves the epithelium proliferation capacity in infected NLRC4-deficient mice. **j** Representative micrographs of cecal tissue sections stained for cell proliferation marker Ki-67 at 72 h p.i. Lu. lumen. Scale bar: 50 µm. **k** Microscopy-based quantification of Ki-67 positive epithelial cells per 63× field of view. **j**, **k** Specific areas without epithelial gaps were chosen. **e**, **f**, **h**, **i**, **k** Each data point represents one mouse. Line at median. Seven mice per group from two independent experiments. Mann–Whitney *U* test (ns—not significant, *p < 0.05, **p < 0.01).

Given that *Nlrc4*^−/−^ mice featured elevated TNF levels at ~48–72 h p.i. (Fig. 5f), and that TNF can compromise cell viability and proliferation,^73,74^ we hypothesized that the observed high TNF concentrations over time may have direct pathological effects on the epithelial barrier. To address this possibility, we exposed 3D enteroids to the range of TNF concentrations measured in vivo. At 18 h post treatment, increasing TNF concentrations lead to a gradual loss of enteroids with pronounced crypt structures (budded enteroids). Instead, more enteroids adopted a spherical shape with a thin epithelial layer (Fig. 6a). A recent study reported similar morphological changes upon TNF treatment and argued that TNF opens intercellular junctions, leading to water influx into the enteroid lumen.^78^ The decreased percentage of enteroids with crypts resembled the late-stage in vivo phenotype in infected NAIP/NLRC4-deficient mice—i.e., decreased average crypt lengths, and lower total crypt numbers (Fig. 6b; compare with e.g., *Nlrc4*^−/−^ 72 h p.i. in Figs. 1a, 5d and S1E). Remarkably, by exposing enteroids to TNF directly upon culture splitting, we detected a pronounced drop in the enteroid regrowth capacity between 10 and 40 ng/ml TNF, as determined by an MTT assay (Fig. 6c). This agreed closely with the mucosal TNF concentration range measured in *Nlrc4*^−/−^ mice at 48–72 h p.i. (i.e., ~20–40 pg/mg tissue; Fig. 5f). Moreover, WT and *Nlrc4*^−/−^ enteroids responded with equal sensitivity to TNF treatment (Fig S9A) and this TNF effect was attributable to Caspase activity (Fig S9B). This provided first proof that the differences in tissue TNF levels between *Nlrc4*^+/−^ and *Nlrc4*^−/−^ mice (Fig. 5f; from ~48 h p.i. onwards) can explain the deleterious effects on the epithelial barrier specifically in the knockout animals.

To investigate if in vivo TNF depletion is sufficient to preserve epithelial barrier integrity in NAIP/NLRC4-deficient mice, we infected *Nlrc4*^−/−^ animals with *S*.Tm for 72 h and injected anti-TNF or isotype control antibodies daily. While luminal colonization was unaffected by anti-TNF treatment (Fig S9C), this treatment resulted in significantly higher *S*.Tm loads in mLN (Fig S9D). Importantly, epithelial barrier integrity was virtually fully restored by anti-TNF treatment (Fig. 6d, e). The total numbers of enterocytes in the tissue also returned to similar levels as those seen in *Nlrc4*^+/−^ mice (Fig. 6d, f; compare to Fig. 1c). H&E staining again revealed a significantly lower degree of epithelial erosion, whereas other inflammatory parameters were broadly similar as in the isotype control-injected animals (Fig. 6g–i). Moreover, anti-TNF treatment restored the numbers of proliferative crypt cells (as judged by Ki-67 staining) to similar levels as in *Nlrc4*^+/−^ control mice (Fig. 6j–k, compare to Fig. 5e). Despite persistently low Lgr5 mRNA levels also in anti-TNF treated animals (Fig S9E), we observed a return to regular villus-crypt architecture with the typical crypt elongation that is associated with epithelium regeneration (Fig. 6j). We speculate that this deficiency may be compensated by other crypt cells acquiring stem cell-like features thus taking over essential stem cell functions.^76^

In conclusion, multiple experimental approaches demonstrate that excessive production of TNF in the mucosa of NAIP/NLRC4-deficient mice can explain the loss of epithelial barrier integrity by 72 h of *S*.Tm infection.

## Discussion

Inflammasomes respond to mucosal infection, but the timing, compartmentalization, and long-term consequences of specific inflammasome pathways remain incompletely understood. Previous work has shown that epithelial NAIP/NLRC4 can promote the expulsion of infected enterocytes and restrict both mucosal tissue and systemic pathogen loads.^37,39,48,49^ Here, we extend these findings and demonstrate that locally acting epithelium-autonomous NAIP/NLRC4 protects the epithelial barrier from collapse during mature stages of the infection (Fig S10).

Epithelial inflammasome activation promotes death of *S*.Tm-infected enterocytes,^29,31,32,37,39,40^ but the salient features have remained a matter of debate. Rauch et al. dissected the mechanism(s) underlying NAIP/NLRC4-dependent enterocyte death with a pure synthetic NAIP5-6 ligand, FlaTox. These experiments suggested that membrane lysis precedes expulsion, and supported a lytic death modality analogous to macrophage pyroptosis, but genetics approaches proved that also apoptotic cell death could in principle occur.^37,50^ Here, we show that live *S*.Tm invasion in vivo elicits NAIP/NLRC4-dependent expulsion of enterocytes that often display classical apoptotic signs—e.g., cleavage of Caspase-3 (Fig. 2e, f). By imaging infections in 3D-, 2D-, and chimeric enteroids, we mapped NAIP/NLRC4-driven cell death features in both space and time (Fig. 3 and S4–6). In light of previous work, our results favor that local, epithelium-autonomous, NAIP/NLRC4 elicits a heterogeneous cell death response that engages both inflammatory (i.e., Caspase-1) and apoptotic Caspases (i.e., Caspase-8 and -3), and causes expulsion of enterocytes that are often initially intact, but lyse on their way into the lumen. Recent studies have shown that a non-canonical Caspase-11 inflammasome can complement this primary epithelial cell death pathway, but requires pro-inflammatory priming for full expression.^29,60^ The relative contribution of NAIP/NLRC4/Caspase-1/8 and Caspase-11/4/5 pathways may also vary between species.^31^ Of note in this context, we found no significant contribution of Caspase-11 to any disease parameter neither in the presence, nor in the absence, of epithelial NAIP/NLRC4 during the first day of *S*.Tm gut infection in mice (refs. ^39,48^ and this study).

The in vivo kinetics experiments further show that epithelial NAIP/NLRC4 promotes the onset of mucosal inflammation (9–18 h p.i.; Fig. 2), but, that lack of this inflammasome in fact leads to exacerbated tissue-destructive inflammation after 72 h (Fig. 5). Our combined results suggest that failure of NAIP/NLRC4 to restrict *S*.Tm tissue loads (through early enterocyte expulsion and/or “protective inflammation”) causes persistent hyperstimulation of pro-inflammatory mucosal circuits that involve BM-derived cell types. This may lead to an inflammatory response with both quantitative (e.g., elevated TNF levels as seen in Fig. 5f) and qualitative differences that tax the regenerative capacity of the epithelial barrier. Importantly, our findings resolve long-standing discrepancies in the earlier literature that have reported either less,^30,37,39^ or more^33,34,49,53,54^ pronounced mucosal inflammation in infected mice lacking NAIP/NLRC4 or downstream Caspases. We argue that these differences stem from the phase of the infection that was analyzed and/or pathogen-specific differences in the time course of the mucosal infection.

Specifically, we find that epithelial barrier destruction in NAIP/NLRC4-deficient animals is linked to excessive TNF levels (Figs. 5–6). Within 24 h p.i., the TNF concentration in cecal tissue increases to intermediate levels (~3–6 pg/mg). While WT mice retain these levels for up to 72 h, *Nlrc4*^−/−^ mice reach up to ~40 pg/mg (Fig. 5f). TNF is known for its context-, receptor-, and dose-dependent pleiotropic effects on the epithelium.^74,79,80^ TNF can (1) trigger a transcriptional response that fuels production of cytokines, mucins, and antimicrobial effectors,^60,80^ (2) modulate intercellular junctions to increase epithelial permeability,^81,82^ and (3) impact epithelial cell migration.^83^ TNF-R2 signaling can (4) promote epithelial cell proliferation,^84^ whereas TNF-R1 signaling (5) induces pronounced intestinal epithelial cell death and shedding.^85,86^ It has become evident that TNF may elicit several modes of epithelial cell death, including apoptosis or necroptosis,^87–89^ and in Gasdermin E-proficient epithelial cells pyroptosis.^90^ The enterocyte cell death response noted in some infected *Nlrc4*^−/−^ mice at 18 h p.i. occurred in the absence of detectable ASC focus formation and Caspase-3/8 activation, but was variably linked to nuclear fragmentation (Fig. 4c, d). At later stages in these mice (i.e., 72 h p.i.), enterocyte cell death appeared heterogeneous, with ASC foci, cleaved Caspase-3 and -8 occasionally detected (Fig S8B). The disrupted tissue architecture however prevented quantitative analysis. In either case, it appears plausible that more than one intracellular pathway contributes to the tissue-destructive enterocyte cell death response in NAIP/NLRC4-deficient animals.

Our enteroid experiments (Fig. 6a–c), supported by earlier work from others,^91,92^ suggest that elevated levels of TNF affect the gut epithelium directly. In vivo, we find TNF to promote enterocyte dislodging and to negatively impact proliferation and stem cell niche maintenance (Fig. 6j–k). These effects in combination suffice to explain the epithelium integrity loss noted in *Nlrc4*^−/−^ mice at 72 h p.i. (Fig. 6d–f). However, we cannot formally rule out that elevated TNF levels also reduce enterocyte migration during gap closure,^83^ or elicit indirect effects (e.g., through macrophages^93^) contributing to epithelium disruption in *S*.Tm-infected NAIP/NLRC4-deficient animals. Still, the simplest explanatory model holds that (1) the absence of an epithelium-autonomous NAIP/NLRC4 defense (2) causes abnormal accumulation of *S*.Tm first in the epithelium and later in the lamina propria compartment. This (3) “forces” BM-derived cell types in the mucosa to hyper-produce TNF in an attempt to restrict the infection. The excessive levels of TNF (4) subsequently act on epithelial TNFR(s) to (5) drive pronounced cell death and suppression of proliferation.

The clinical use of TNF-neutralizing antibodies has been a major breakthrough benefitting a significant fraction of patients with for example inflammatory bowel disease.^94^ However, the underlying mechanisms remain incompletely understood. Besides depleting soluble TNF, it has for instance been reported that anti-TNF targeting of membrane-bound TNF induces T cell apoptosis, and that anti-TNF can interact with the Fc receptors on macrophages, triggering their differentiation to an M2-like state.^95^ These various modes of action may explain the variable success rates among anti-TNF treatment regimens.^95^ Our work offers an appealing murine experimental system to better understand the modes of action for TNF in vivo, and to tease apart the cellular and molecular basis for anti-TNF treatment approaches. In contrast to other murine colitis models, the herein used *S*.Tm infection model follows precise and reproducible kinetics, which can help resolve TNF-dependent and independent effects on the mucosa during the stage-wise progression of gut inflammatory disease.

In summary, we have here mapped the epithelial NAIP/NLRC4 response across acute and late-stage *S*.Tm gut infection in vivo, and in epithelial enteroids. Our work uncovers a central role of epithelium-autonomous NAIP/NLRC4 in eliciting a fine-tuned early response to the infection, thereby preventing the demise of the epithelial barrier, due to an overshooting pro-inflammatory TNF response in the intestinal mucosa.

## Methods

### Salmonella, strains, plasmids, and culture conditions

*Salmonella* Typhimurium SL1344 (SB300, SmR) was used as WT. The plasmid pM975 (*pssaG-*GFPmut2) has been previously used.^15^ For in vivo and tissue culture infections, *S*.Tm was cultured in LB/0.3 M NaCl (Sigma-Aldrich) with appropriate antibiotics for 12 h before sub-culturing at a 1:20 dilution for 4 h in the same broth without antibiotics. For in vivo infections, the inoculum was reconstituted in PBS (BioConcept), and for tissue culture infections in either DMEM/F12 (Gibco) supplemented with 3% FBS (Gibco), or in complete mouse Intesticult (STEMCELL).

### Mice and in vivo infections

Specific pathogen-free mice were maintained in individually ventilated cages of the ETH Zurich mouse facility (EPIC and RCHCI). WT mice, C57BL/6, originated from Charles River (Sulzfeld, Germany). The following transgenic and knockout mouse lines, all in C57BL/6 background, were used: *Nlrc4*^−/−^ (B6.C2-Nlrc4tm1Vmd,^55^), *Naip1–6*^*Δ/ΔIEC*^,^57^ *RFP*^*IEC*^,^96^ *Atg7* ^*Δ/ΔIEC*^ (this study, generated by crossing *VilCre* mice^97^ with *Atg7*^*fl/fl*^ mice,^98^ *Casp11*^−/−^ (B6.Casp11^tm1^,^99^), *Tnf*^−/−^ (B6.129-Tnf^tm1Ljo^,^100^), *Nlrc4*^−/−^*Nlrp3*^−/−^,^48^ *Nlrc4*^−/−^*Casp11*^−/−^,^48^ and *RFP*^*IEC*^*Naip1–6*^*Δ/ΔIEC*^ (this study, generated by crossing the *RFP*^*IEC*^ and *Naip1–6*^*Δ/ΔIEC*^ lines). Mouse lines were genotyped by PCR and heterozygous/homozygous littermates were used for infection experiments. Eight- to twelve-week-old mice were infected according to the streptomycin mouse model.^56^ Briefly, mice were orally pretreated with 25 mg streptomycin sulfate (Sm; Applichem) 1 day prior to infection and orally infected with ~5 × 10^7^ CFU *S*.Tm by oral gavage. Bacterial burdens were analyzed by CFU plating assays on MacConkey agar (Oxoid) with 50 µg/ml streptomycin. For cecal tissue loads, the cecum was opened up, the content removed, the tissue incubated in PBS/400 µg/ml gentamycin for 30 min, and subsequently extensively washed six times in PBS before homogenization (TissueLyser Qiagen) and plating. For BM chimeras, *Nlrc4*^−/−^ recipient mice were irradiated (1000 Rad, 14 min) and received 5 × 10^6^ BM cells via tail vein from *Tnf*^+/−^ or *Tnf*^−/−^ donor mice, respectively. Mice were kept on Borgal (Vererinaria AG) for 3 weeks and infected 8–10 weeks after reconstitution. For in vivo depletion, anti-IFNγ (Bio X Cell, R4-6A2, 500 µg twice daily), or anti-TNF (Bio X Cell, XT3.11, for Fig. 4 and S5: once 500 µg during Sm-pretreatment, for Fig. 6 and S7: 200 µg daily starting 1 day prior to infection) antibodies were injected i.p. Anti-trinitrophenol (Bio X Cell, TNP6A7, 500 µg twice daily), or anti-horseradish peroxidase (Bio X Cell, HRPN, Fig. 4 and S5: once 500 µg during Sm-pretreatment) were used as isotype controls. All animal experiments were approved by the Kantonales Veterinäramt Zürich (licences 222/2013 and 193/2016).

### Murine 3D intestinal epithelial enteroids and infections

Murine jejunal epithelial enteroids were established and maintained in complete mouse IntestiCult (STEMCELL) supplemented with PenStrep (Gibco), as previously described.^60^ Briefly, mouse jejunum was cut in 2 mm pieces, extensively washed in ice-cold PBS, incubated in Gentle cell dissociation reagent (STEMCELL) on a rocking table (20 rpm, 15 min, RT) and intestinal crypts were extracted by mechanical shearing in PBS/0.1%BSA (Chemie Brunschwig AG). Crypts were passed through a 70 µm cell strainer, washed and embedded in 50 µl Matrigel (Corning) domes (250–1000 crypts per dome) covered with 600 µl complete mouse IntestiCult. The medium was exchanged every 2–3 days and the cultures were split every 5–7 days by mechanical shearing in Gentle cell dissociation reagent and re-embedding in 50 µl Matrigel domes at a 1:2 to 1:4 splitting ratio. Stable cultures were cryopreserved and thawed for experimentation. All experiments were performed after at least 2 weeks of culture maintenance. Chimeric enteroids were established by mixing fragments from the indicated enteroids 1:1 at the splitting step and re-embedding the fragment suspension at high density (~400 enteroids/50 µl dome) in Matrigel domes. For *S*.Tm bulk infections, 50 µl Matrigel domes containing ~100 enteroids were dissolved in ice-cold DMEM/F12/3%FBS 2–4 days after culture splitting. The enteroid suspension was centrifuged (300 g, 5 min, 4 °C) and re-suspended in pre-warmed DMEM/F12/3%FBS before adding *S*.Tm at MOI ~100, assuming ~1000 epithelial cells per enteroid (estimation based on confocal Z-stack micrographs). After 40 min of infection (37 °C, 5% CO_2_), extracellular *S*.Tm were killed by incubation (15 min, 37 °C) in DMEM/F12/3%FCS supplemented with 100 µg/ml gentamycin (Axon Lab AG). Enteroids were re-suspended in complete mouse IntestiCult/25 µg/ml gentamycin and seeded in 60% Matrigel domes (25 µl domes for 8-well chambered slides, 50 µl domes for 24-well plates). Complete IntestiCult/25 µg/ml gentamycin was added to solidified domes and enteroids were incubated (37 °C, 5% CO_2_) until microscopy analysis or CFU plating assays at the indicated time points. For plating assays, enteroids were dissolved in ice-cold PBS/3%FBS at 21 h p.i., washed three times with PBS/3%FBS and lysed in 0.1% sodium deoxycholate (Sigma-Aldrich). The lysates containing intracellular bacteria were serially diluted and plated on LB agar containing appropriate antibiotics. For TNF treatment, enteroids were either treated 3 days post culture splitting, or directly after culture splitting, with the indicated concentration of recombinant TNF (Peprotech). MTT assay was performed as described previously.^101^

### Murine enteroid-derived 2D epithelial monolayers, infections, and time-lapse microscopy

Conditions for single origin and chimeric 2D monolayers are validated elsewhere (Samperio Ventayol et al. 2021, in revision). Briefly, matrigel-embedded enteroids were treated with 3 µM CHIR99021 and 1 mM valproic acid (VPA) in complete mouse IntestiCult for 4–7 days, changing the medium every 2–3 days. Enteroids were dissociated using Gentle Cell Dissociation Reagent (STEMCELL) and further disrupted mechanically with a G25 needle. For chimeric monolayers, epithelial cell suspensions from WT and *RFP*^*IEC*^*Naip1–6*^*Δ/Δ*^ enteroids were mixed in equal proportions at this stage. 150,000 cells∙cm^−2^ were seeded on top of a polymerized collagen-I hydrogel (as described in^102^) over Poly-Lysine coated 8-well chambers, and kept in IntestiCult supplemented with 3 µM CHIR99021, 1 mM VPA and 10 µM Y-27632 for 24 h, thereafter omitting these additives and changing the culture medium to complete mouse IntestiCult with PenStrep. After 72–96 h of establishment, monolayers were washed with DMEM/F12 and the medium was exchanged for antibiotics-free medium 2–6 h before infection. Draq7 staining solution (Biostatus) was added 15 min before infection, together with Z-VAD-FMK when specified, at final concentrations of 0.3–1.5 and 50 µM, respectively. The corresponding amount of bacteria was dissolved in complete mouse IntestiCult to a final MOI of 2–20 as indicated and added directly to the wells while the microscope chamber was maintained at 37 °C in a 5% CO_2_ moisturized atmosphere. Time-lapses every 20 s, or 2–3 min, were acquired with a custom-built microscope, based on a Nikon Eclipse Ti2 core fitted with a 60×/0.7 or 40×/0.6 Plan Apo-Lambda air objectives (Nikon), a X-Light-V2-LFOV spinning disk module (Crest), and a Prime 95B 25 mm camera (Photometrics) directly upon infection. For Draq7 quantification, the single cell expulsion starting time points were selected based on the DIC channel. Mean Draq7 intensity was measured in a 14.5 µm^2^ circle positioned manually around the nucleus in each time-lapse frame (every 20 s) before and after the expulsion starting time point, using ImageJ.

### Immunofluorescence staining, wide field- and confocal microscopy

Mouse cecal tissue was fixed with 4% paraformaldehyde, saturated in 20% sucrose, and flash frozen in optimal cutting temperature compound (OCT, Tissue-Tek). Samples were stored at −80 °C until further analysis. 10–20 µm thick cryosections were cut and mounted on glass slides (Superfrost+ +, Thermo Scientific). For fluorescence staining, air-dried cryosections were rehydrated with PBS and treated with PBS/0.5% Tx-100 to permeabilize cells. PBS/10% normal goat serum (Reactolab SA) was used for blocking. Enteroid-derived 2D monolayers were washed in PBS, fixed with 2% paraformaldehyde, permeabilized with PBS/0.5% Tx-100, and blocked in PBS/1% BSA. Stainings included the antibodies α-EpCam/CD326 (clone G8.8, Biolegend), α-ICAM-1/CD54 (clone 3E2, Becton Dickinson), α-ASC (N-15, Santa Cruz Biotechnology), α-cleaved Caspase-3 (#9661, Cell Signaling Technology), α-cleaved Caspase-8 (#9496, Cell Signaling Technology), α-Ki-67 (ab15580, Abcam Biochemicals), or α-*S*.Tm-LPS (O-antigen group B factor 4-5, Difco), combined with appropriate secondary antibodies, i.e., α-hamster-Cy3 (Jackson), α-rabbit-AlexaFluor488 (Abcam Biochemicals), α-rabbit-Cy3 (Bethyl Laboratories), α-rat-FITC (Jackson), α-rat-Cy3 (Jackson), α-rat-Cy5 (Jackson) and fluorescent probes, i.e., CruzFluor488-conjugated Phalloidin (Santa Cruz Biotechnology), TRITC-conjugated Phalloidin (Fluoprobes), AlexaFluor647-conjugated Phalloidin (Molecular Probes), AlexaFluor647-conjugated wheat-germ agglutinin (Molecular Probes), and/or DAPI (Sigma-Aldrich). Stained tissue sections were covered with a glass slip using Mowiol (VWR International AG) and imaged the following day. For confocal microscopy, we used either a Zeiss Axiovert 200 m microscope with 10–100× objectives, a spinning disc confocal lased unit (Visitron), and an Evolve 512 EMCCD camera (Photometrics) or a Zeiss LSM 700, with 10–100× objectives. For wide field microscopy, we used a Nikon Ti Eclipse, with 10–60× objectives. Images were processed or analyzed with Visiview (Visitron), Zeiss ZEN, NIS-Elements Advanced Research software, and/or ImageJ. Manual microscopy quantifications of the parameters indicated were done blindly on at least three non-consecutive sections per mouse. Epithelial gaps in fluorescence microscopy were defined as mucosal regions where lamina propria cells were directly exposed to the gut lumen. Epithelial erosions in H&E-stained cecal tissue sections were defined as mucosal regions where a continuous epithelium layer was absent. Dislodging cells were defined as cells associated with epithelium that showed signs of expulsion. In contrast, dislodged cells were defined as cells clearly separated from the epithelium and residing in the lumen.

### Histology

Mouse cecal tissue was directly frozen in OCT, cut in 5 µm cross-sections, air-dried and stained with hematoxylin and eosin. Histology score was enumerated blindly as described in detail elsewhere.^56^ A score of 0 represents an intact intestine without any signs of inflammation, 1–2 represents minimal signs of inflammation, 3–4 represents slight inflammation, 5–8 represents moderate inflammation and 9–13 represents profound inflammation.

### TNF ELISA

Cecal tissue samples were extensively washed in PBS before homogenizing in PBS/0.5%Tergitol/0.5%BSA (Sigma-Aldrich, Chemie Brunschwig AG) supplemented with protease inhibitor cocktail (Roche). TNF concentration was determined with TNF alpha Mouse ELISA Kit High Sensitivity (Invitrogen) according to the manufacturer’s protocol.

### RT-qPCR

Cecal content was removed from cecal tissue samples, these were flash frozen in RNAlater (Invitrogen) and stored at −80 °C until further processed. RNA was isolated using RNeasy Mini Kit (Qiagen) and reverse transcribed employing RT^2^ HT First Strand cDNA Kit (Qiagen). qPCR was performed with FastStart Universal SYBR Green Master reagents (Roche) on a QuantStudio 7 Flex FStepOne Plus Cycler. Primers were purchased as validated primer assays from Qiagen, except for those primer pairs detecting *Caspase-3*, *Caspase-8*, *Lgr5*, and *Acl2* transcripts (listed in Table 1).

**Table 1:** Custom-designed primers used in this study.

Gene	Forward primer sequence	Reverse primer sequence
Mouse *Caspase-3*	TGACTGGAAAGCCGAAACTC	AGCCTCCACCGGTATCTTCT
Mouse *Caspase-8*	ATGGCTACGGTGAAGAACTGCG	TAGTTCACGCCAGTCAGGATGC
Mouse *Lgr5*	ACCCGCCAGTCTCCTACATC	GCATCTAGGCGCAGGGATTG
Mouse *Ascl2*	GAGAGCTAAGCCCGATGGAG	CCAGGGATGCAGCTTAGGG

### Statistical analysis

Where applicable, statistical significance was assessed by the Mann–Whitney *U* test, Multiple t-test using Bonferroni–Dunn method, one-way or two-way ANOVA with Tukey HSD as indicated in the figure legends.

## Supplementary information

Supplementary Information

## References

[1] Beumer J, Clevers H (2016). Regulation and plasticity of intestinal stem cells during homeostasis and regeneration. Development.

[2] Koch S, Nusrat A (2012). The life and death of epithelia during inflammation: lessons learned from the gut. Annu. Rev. Pathol..

[3] Ingram JP (2018). A nonpyroptotic IFN-gamma-triggered cell death mechanism in nonphagocytic cells promotes salmonella clearance in vivo. J. Immunol..

[4] Marchiando AM (2011). The epithelial barrier is maintained by in vivo tight junction expansion during pathologic intestinal epithelial shedding. Gastroenterology.

[5] Nava P (2010). Interferon-gamma regulates intestinal epithelial homeostasis through converging beta-catenin signaling pathways. Immunity.

[6] Cooper HS, Murthy SN, Shah RS, Sedergran DJ (1993). Clinicopathologic study of dextran sulfate sodium experimental murine colitis. Lab Investig..

[7] Patterson AM, Watson AJM (2017). Deciphering the complex signaling systems that regulate intestinal epithelial cell death processes and shedding. Front Immunol..

[8] Hausmann A, Hardt WD (2019). The Interplay between Salmonella enterica Serovar Typhimurium and the intestinal mucosa during oral infection. Microbiol. Spectr..

[9] Furter M, Sellin ME, Hansson GC, Hardt WD (2019). Mucus architecture and near-surface swimming affect distinct salmonella typhimurium infection patterns along the murine intestinal tract. Cell Rep..

[10] Stecher B (2008). Motility allows S. Typhimurium to benefit from the mucosal defence. Cell Microbiol..

[11] Stecher B (2004). Flagella and chemotaxis are required for efficient induction of Salmonella enterica serovar Typhimurium colitis in streptomycin-pretreated mice. Infect. Immun..

[12] Fattinger SA (2020). Salmonella Typhimurium discreet-invasion of the murine gut absorptive epithelium. PLoS Pathog..

[13] Galan JE, Curtiss R (1989). Cloning and molecular characterization of genes whose products allow Salmonella typhimurium to penetrate tissue culture cells. Proc. Natl Acad. Sci. USA.

[14] Hapfelmeier S (2004). Role of the Salmonella pathogenicity island 1 effector proteins SipA, SopB, SopE, and SopE2 in Salmonella enterica subspecies 1 serovar Typhimurium colitis in streptomycin-pretreated mice. Infect. Immun..

[15] Hapfelmeier S (2005). The Salmonella pathogenicity island (SPI)-2 and SPI-1 type III secretion systems allow Salmonella serovar typhimurium to trigger colitis via MyD88-dependent and MyD88-independent mechanisms. J. Immunol..

[16] Santos RL, Zhang S, Tsolis RM, Baumler AJ, Adams LG (2002). Morphologic and molecular characterization of Salmonella typhimurium infection in neonatal calves. Vet. Pathol..

[17] Zhang K (2018). Minimal SPI1-T3SS effector requirement for Salmonella enterocyte invasion and intracellular proliferation in vivo. PLoS Pathog..

[18] Ermund A, Schutte A, Johansson ME, Gustafsson JK, Hansson GC (2013). Studies of mucus in mouse stomach, small intestine, and colon. I. Gastrointestinal mucus layers have different properties depending on location as well as over the Peyer’s patches. Am. J. Physiol. Gastrointest. Liver Physiol..

[19] Johansson ME (2008). The inner of the two Muc2 mucin-dependent mucus layers in colon is devoid of bacteria. Proc. Natl Acad. Sci. USA.

[20] Muniz LR, Knosp C, Yeretssian G (2012). Intestinal antimicrobial peptides during homeostasis, infection, and disease. Front. Immunol..

[21] Maier L (2014). Granulocytes impose a tight bottleneck upon the gut luminal pathogen population during Salmonella typhimurium colitis. PLoS Pathog..

[22] Muller AA (2016). An NK cell perforin response elicited via IL-18 controls mucosal inflammation kinetics during salmonella gut infection. PLoS Pathog..

[23] Rydstrom A, Wick MJ (2007). Monocyte recruitment, activation, and function in the gut-associated lymphoid tissue during oral Salmonella infection. J. Immunol..

[24] Koscso B (2020). Gut-resident CX3CR1(hi) macrophages induce tertiary lymphoid structures and IgA response in situ. Sci. Immunol..

[25] Moor K (2017). High-avidity IgA protects the intestine by enchaining growing bacteria. Nature.

[26] Fattinger SA, Sellin ME, Hardt WD (2020). Epithelial inflammasomes in the defense against Salmonella gut infection. Curr. Opin. Microbiol..

[27] Broz P (2010). Redundant roles for inflammasome receptors NLRP3 and NLRC4 in host defense against Salmonella. J. Exp. Med..

[28] Carvalho FA (2012). Cytosolic flagellin receptor NLRC4 protects mice against mucosal and systemic challenges. Mucosal Immunol..

[29] Crowley SM (2020). Intestinal restriction of Salmonella Typhimurium requires caspase-1 and caspase-11 epithelial intrinsic inflammasomes. PLoS Pathog..

[30] Franchi L (2012). NLRC4-driven production of IL-1beta discriminates between pathogenic and commensal bacteria and promotes host intestinal defense. Nat. Immunol..

[31] Holly MK (2020). Salmonella enterica infection of murine and human enteroid-derived monolayers elicits differential activation of epithelial-intrinsic inflammasomes. Infect. Immun..

[32] Knodler LA (2010). Dissemination of invasive Salmonella via bacterial-induced extrusion of mucosal epithelia. Proc. Natl Acad. Sci. USA.

[33] Lai MA (2013). Innate immune detection of flagellin positively and negatively regulates salmonella infection. PLoS ONE.

[34] Lara-Tejero M (2006). Role of the caspase-1 inflammasome in Salmonella typhimurium pathogenesis. J. Exp. Med..

[35] Miao EA (2010). Caspase-1-induced pyroptosis is an innate immune effector mechanism against intracellular bacteria. Nat. Immunol..

[36] Muller AJ (2009). The S. Typhimurium effector SopE induces caspase-1 activation in stromal cells to initiate gut inflammation. Cell Host Microbe.

[37] Rauch I (2017). NAIP-NLRC4 inflammasomes coordinate intestinal epithelial cell expulsion with eicosanoid and IL-18 release via activation of caspase-1 and -8. Immunity.

[38] Raupach B, Peuschel SK, Monack DM, Zychlinsky A (2006). Caspase-1-mediated activation of interleukin-1beta (IL-1beta) and IL-18 contributes to innate immune defenses against Salmonella enterica serovar Typhimurium infection. Infect. Immun..

[39] Sellin ME (2014). Epithelium-intrinsic NAIP/NLRC4 inflammasome drives infected enterocyte expulsion to restrict Salmonella replication in the intestinal mucosa. Cell Host Microbe.

[40] Knodler LA (2014). Noncanonical inflammasome activation of caspase-4/caspase-11 mediates epithelial defenses against enteric bacterial pathogens. Cell Host Microbe.

[41] Sellin ME, Maslowski KM, Maloy KJ, Hardt WD (2015). Inflammasomes of the intestinal epithelium. Trends Immunol..

[42] Winsor N, Krustev C, Bruce J, Philpott DJ, Girardin SE (2019). Canonical and noncanonical inflammasomes in intestinal epithelial cells. Cell Microbiol.

[43] Reyes Ruiz VM (2017). Broad detection of bacterial type III secretion system and flagellin proteins by the human NAIP/NLRC4 inflammasome. Proc. Natl Acad. Sci. USA.

[44] Yang J, Zhao Y, Shi J, Shao F (2013). Human NAIP and mouse NAIP1 recognize bacterial type III secretion needle protein for inflammasome activation. Proc. Natl Acad. Sci. USA.

[45] Zhao Y (2011). The NLRC4 inflammasome receptors for bacterial flagellin and type III secretion apparatus. Nature.

[46] Rauch I (2016). NAIP proteins are required for cytosolic detection of specific bacterial ligands in vivo. J. Exp. Med..

[47] Zhao Y (2016). Genetic functions of the NAIP family of inflammasome receptors for bacterial ligands in mice. J. Exp. Med..

[48] Hausmann A (2020). Intestinal epithelial NAIP/NLRC4 restricts systemic dissemination of the adapted pathogen Salmonella Typhimurium due to site-specific bacterial PAMP expression. Mucosal Immunol.

[49] Nordlander S, Pott J, Maloy KJ (2014). NLRC4 expression in intestinal epithelial cells mediates protection against an enteric pathogen. Mucosal Immunol..

[50] Van Opdenbosch N (2017). Caspase-1 engagement and TLR-induced c-FLIP expression suppress ASC/caspase-8-dependent apoptosis by inflammasome sensors NLRP1b and NLRC4. Cell Rep..

[51] Sellin ME, Muller AA, Hardt WD (2018). Consequences of epithelial inflammasome activation by bacterial pathogens. J. Mol. Biol..

[52] Iyer N (2020). Epithelium intrinsic vitamin A signaling co-ordinates pathogen clearance in the gut via IL-18. PLoS Pathog..

[53] Mitchell PS (2020). NAIP-NLRC4-deficient mice are susceptible to shigellosis. Elife.

[54] Hefele M (2018). Intestinal epithelial Caspase-8 signaling is essential to prevent necroptosis during Salmonella Typhimurium induced enteritis. Mucosal Immunol..

[55] Mariathasan S (2004). Differential activation of the inflammasome by caspase-1 adaptors ASC and Ipaf. Nature.

[56] Barthel M (2003). Pretreatment of mice with streptomycin provides a Salmonella enterica serovar Typhimurium colitis model that allows analysis of both pathogen and host. Infect. Immun..

[57] Allam R (2015). Epithelial NAIPs protect against colonic tumorigenesis. J. Exp. Med..

[58] Broz P, von Moltke J, Jones JW, Vance RE, Monack DM (2010). Differential requirement for Caspase-1 autoproteolysis in pathogen-induced cell death and cytokine processing. Cell Host Microbe.

[59] Van Opdenbosch N (2014). Activation of the NLRP1b inflammasome independently of ASC-mediated caspase-1 autoproteolysis and speck formation. Nat. Commun..

[60] Hausmann A (2020). Germ-free and microbiota-associated mice yield small intestinal epithelial organoids with equivalent and robust transcriptome/proteome expression phenotypes. Cell Microbiol..

[61] Sato T (2009). Single Lgr5 stem cells build crypt-villus structures in vitro without a mesenchymal niche. Nature.

[62] Bierschenk D (2019). The Salmonella pathogenicity island-2 subverts human NLRP3 and NLRC4 inflammasome responses. J. Leukoc. Biol..

[63] Birmingham CL, Smith AC, Bakowski MA, Yoshimori T, Brumell JH (2006). Autophagy controls Salmonella infection in response to damage to the Salmonella-containing vacuole. J. Biol. Chem..

[64] Klose CS (2013). A T-bet gradient controls the fate and function of CCR6-RORgammat+ innate lymphoid cells. Nature.

[65] Mastroeni P, Skepper JN, Hormaeche CE (1995). Effect of anti-tumor necrosis factor alpha antibodies on histopathology of primary Salmonella infections. Infect. Immun..

[66] Pham THM (2020). Salmonella-driven polarization of granuloma macrophages antagonizes TNF-mediated pathogen restriction during persistent infection. Cell Host Microbe.

[67] Qu Y (2016). NLRP3 recruitment by NLRC4 during Salmonella infection. J. Exp. Med..

[68] Songhet P (2011). Stromal IFN-gammaR-signaling modulates goblet cell function during Salmonella Typhimurium infection. PLoS One.

[69] Xu Y (2019). A bacterial effector reveals the V-ATPase-ATG16L1 axis that initiates xenophagy. Cell.

[70] Swanson KV, Deng M, Ting JP (2019). The NLRP3 inflammasome: molecular activation and regulation to therapeutics. Nat. Rev. Immunol..

[71] Matikainen S, Nyman TA, Cypryk W (2020). Function and regulation of noncanonical caspase-4/5/11 inflammasome. J. Immunol..

[72] Ingram JP, Brodsky IE, Balachandran S (2017). Interferon-gamma in Salmonella pathogenesis: new tricks for an old dog. Cytokine.

[73] Weinlich R, Oberst A, Beere HM, Green DR (2017). Necroptosis in development, inflammation and disease. Nat. Rev. Mol. Cell Biol..

[74] Ruder B, Atreya R, Becker C (2019). Tumour necrosis factor alpha in intestinal homeostasis and gut related diseases. Int. J. Mol. Sci..

[75] Garside P, Bunce C, Tomlinson RC, Nichols BL, Mowat AM (1993). Analysis of enteropathy induced by tumour necrosis factor alpha. Cytokine.

[76] Schmitt M (2018). Paneth cells respond to inflammation and contribute to tissue regeneration by acquiring stem-like features through SCF/c-Kit signaling. Cell Rep..

[77] Gerdes J, Schwab U, Lemke H, Stein H (1983). Production of a mouse monoclonal antibody reactive with a human nuclear antigen associated with cell proliferation. Int J. Cancer.

[78] Hahn S (2017). Organoid-based epithelial to mesenchymal transition (OEMT) model: from an intestinal fibrosis perspective. Sci. Rep..

[79] Kalliolias GD, Ivashkiv LB (2016). TNF biology, pathogenic mechanisms and emerging therapeutic strategies. Nat. Rev. Rheumatol..

[80] Leppkes M, Roulis M, Neurath MF, Kollias G, Becker C (2014). Pleiotropic functions of TNF-alpha in the regulation of the intestinal epithelial response to inflammation. Int Immunol..

[81] Bruewer M (2003). Proinflammatory cytokines disrupt epithelial barrier function by apoptosis-independent mechanisms. J. Immunol..

[82] Wang F (2005). Interferon-gamma and tumor necrosis factor-alpha synergize to induce intestinal epithelial barrier dysfunction by up-regulating myosin light chain kinase expression. Am. J. Pathol..

[83] Corredor J (2003). Tumor necrosis factor regulates intestinal epithelial cell migration by receptor-dependent mechanisms. Am. J. Physiol. Cell Physiol..

[84] Mizoguchi E (2002). Role of tumor necrosis factor receptor 2 (TNFR2) in colonic epithelial hyperplasia and chronic intestinal inflammation in mice. Gastroenterology.

[85] Grabinger T (2017). Inhibitor of apoptosis protein-1 regulates tumor necrosis factor-mediated destruction of intestinal epithelial cells. Gastroenterology.

[86] Piguet PF, Vesin C, Guo J, Donati Y, Barazzone C (1998). TNF-induced enterocyte apoptosis in mice is mediated by the TNF receptor 1 and does not require p53. Eur. J. Immunol..

[87] Gunther C (2011). Caspase-8 regulates TNF-alpha-induced epithelial necroptosis and terminal ileitis. Nature.

[88] Pott J, Kabat AM, Maloy KJ (2018). Intestinal epithelial cell autophagy is required to protect against TNF-induced apoptosis during chronic colitis in mice. Cell Host Microbe.

[89] Takahashi N (2014). RIPK1 ensures intestinal homeostasis by protecting the epithelium against apoptosis. Nature.

[90] Wang Y (2017). Chemotherapy drugs induce pyroptosis through caspase-3 cleavage of a gasdermin. Nature.

[91] Grabinger T (2014). Ex vivo culture of intestinal crypt organoids as a model system for assessing cell death induction in intestinal epithelial cells and enteropathy. Cell Death Dis..

[92] Schreurs R (2019). Human fetal TNF-alpha-cytokine-producing CD4(+) effector memory T cells promote intestinal development and mediate inflammation early in life. Immunity.

[93] Wynn TA, Vannella KM (2016). Macrophages in tissue repair, regeneration, and fibrosis. Immunity.

[94] Neurath MF (2014). Cytokines in inflammatory bowel disease. Nat. Rev. Immunol..

[95] Levin AD, Wildenberg ME, van den Brink GR (2016). Mechanism of action of anti-TNF therapy in inflammatory bowel disease. J. Crohns Colitis.

[96] Muller AJ (2012). Salmonella gut invasion involves TTSS-2-dependent epithelial traversal, basolateral exit, and uptake by epithelium-sampling lamina propria phagocytes. Cell Host Microbe.

[97] Madison BB (2002). Cis elements of the villin gene control expression in restricted domains of the vertical (crypt) and horizontal (duodenum, cecum) axes of the intestine. J. Biol. Chem..

[98] Komatsu M (2005). Impairment of starvation-induced and constitutive autophagy in Atg7-deficient mice. J. Cell Biol..

[99] Kayagaki N (2011). Non-canonical inflammasome activation targets caspase-11. Nature.

[100] Marino MW (1997). Characterization of tumor necrosis factor-deficient mice. Proc. Natl Acad. Sci. USA.

[101] Grabinger T, Delgado E, Brunner T (2016). Analysis of cell death induction in intestinal organoids in vitro. Methods Mol. Biol..

[102] Wang Y (2017). Self-renewing monolayer of primary colonic or rectal epithelial cells. Cell Mol. Gastroenterol. Hepatol..

[103] Dolowschiak T (2016). IFN-gamma hinders recovery from mucosal inflammation during antibiotic therapy for salmonella gut infection. Cell Host Microbe.

