# Supplementary material for: Epithelium-autonomous NAIP/NLRC4 prevents TNF-driven inflammatory destruction of the gut epithelial barrier in Salmonella-infected mice
Source: Mucosal Immunol. 2021 Mar 17;14(3):615–29. doi: 10.1038/s41385-021-00381-y (PMC8075861; doi:10.1038/s41385-021-00381-y)
Supplement: Supplementary file 1 — Supplementary Information [file 41385_2021_381_MOESM1_ESM.pdf]

Supplementary information

Epithelium-autonomous NAIP/NLRC4 prevents TNF-driven inflammatory destruction of the gut epithelial barrier in *Salmonella* infected mice

Stefan A. Fattinger[1,2], Petra Geiser[2], Pilar Samperio Ventayol[2], Maria Letizia Di Martino[2], Markus Furter[1], Boas Felmy[1], Erik Bakkeren[1], Annika Hausmann[1], Manja Barthel-Scherrer[1], Ersin Gül[1], Wolf-Dietrich Hardt[1]*, Mikael E. Sellin[1,2]*

Affiliations:

[1]Institute of Microbiology, Department of Biology, ETH Zurich, Zurich, Switzerland

[2]Science for Life Laboratory, Department of Medical Biochemistry and Microbiology, Uppsala University, Uppsala, Sweden

*Correspondence: WDH; hardt@micro.biol.ethz.ch, or MES; mikael.sellin@imbim.uu.se

Figure S1 - Supplementary figure to Fig1

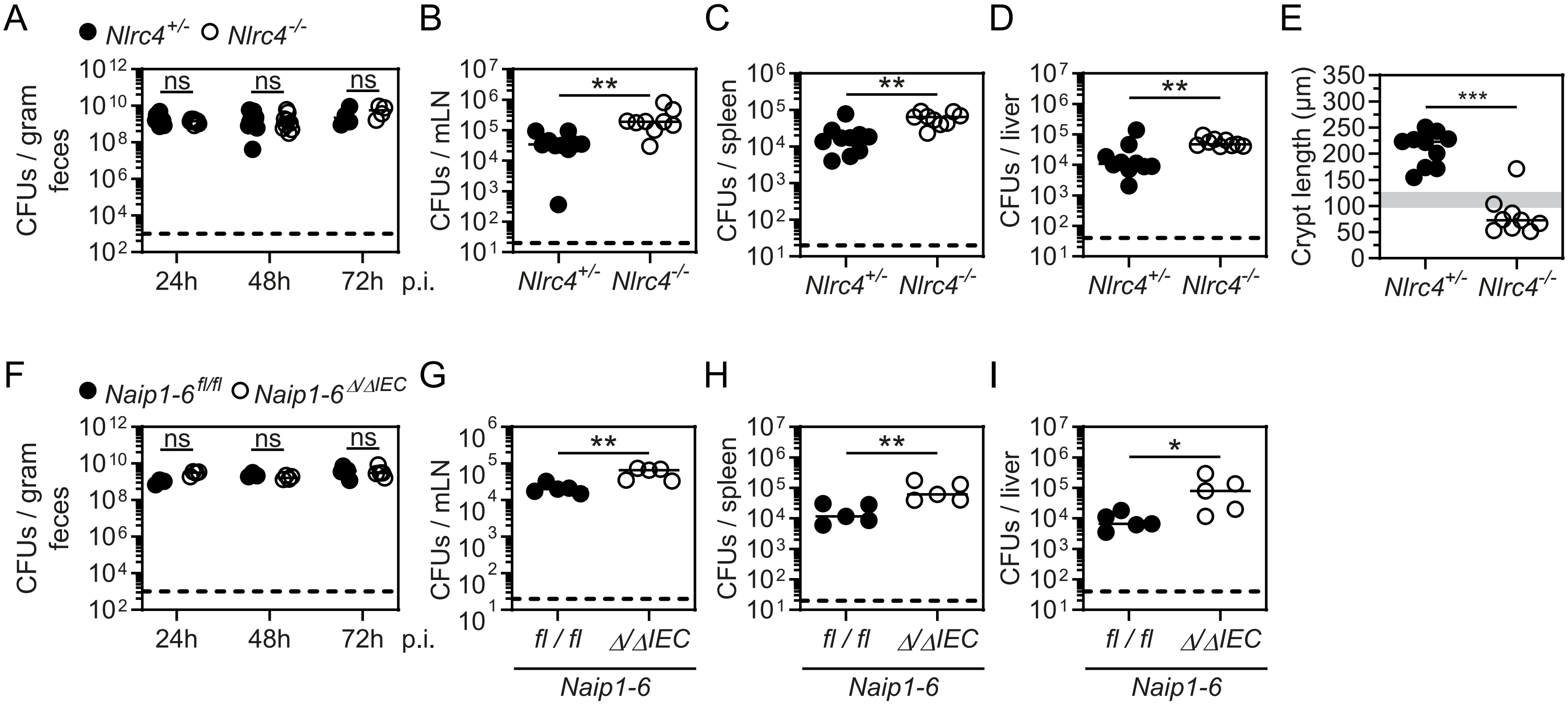

Figure S1 (supporting data for Figure 1)

(**A**) *S.*Tm CFU counts in feces over time in *Nlrc4*$^{+/-}$ and *Nlrc4*$^{-/-}$ littermates. (**B-D**) Systemic *S.*Tm CFU counts at 72h p.i. in *Nlrc4*$^{+/-}$ and *Nlrc4*$^{-/-}$ littermates. (**B**) *S.*Tm CFU counts in mesenteric lymph nodes. (**C**) *S.*Tm CFU counts in spleen. (**D**) *S.*Tm CFU counts in liver. (**E**) Microscopy-based quantification of crypt length at 72h p.i. in *Nlrc4*$^{+/-}$ and *Nlrc4*$^{-/-}$ littermates. Grey area represents steady state crypt length (see Fig S2F) (**F**) *S.*Tm CFU counts in feces over time in *Naip1-6*$^{fl/fl}$ and *Naip1-6*$^{\Delta/\Delta IEC}$ littermates. (**G-I**) Systemic *S.*Tm CFU counts at 72h p.i. in *Naip1-6*$^{fl/fl}$ and *Naip1-6*$^{\Delta/\Delta IEC}$ littermates. (**G**) *S.*Tm CFU counts in mesenteric lymph nodes. (**H**) *S.*Tm CFU counts in spleen. (**I**) *S.*Tm CFU counts in liver. Dashed lines represent detection limit and each data point represents one mouse. Line at median. 5-10 mice per group from ≥2 independent experiments for each comparison. Mann-Whitney U-test (ns - not significant, *p<0.05, **p<0.01, ***<0.001).

Figure S2 - *Nlrc4*$^{+/-}$ vs. *Nlrc4*$^{-/-}$ - steady state parameters in the cecal mucosa

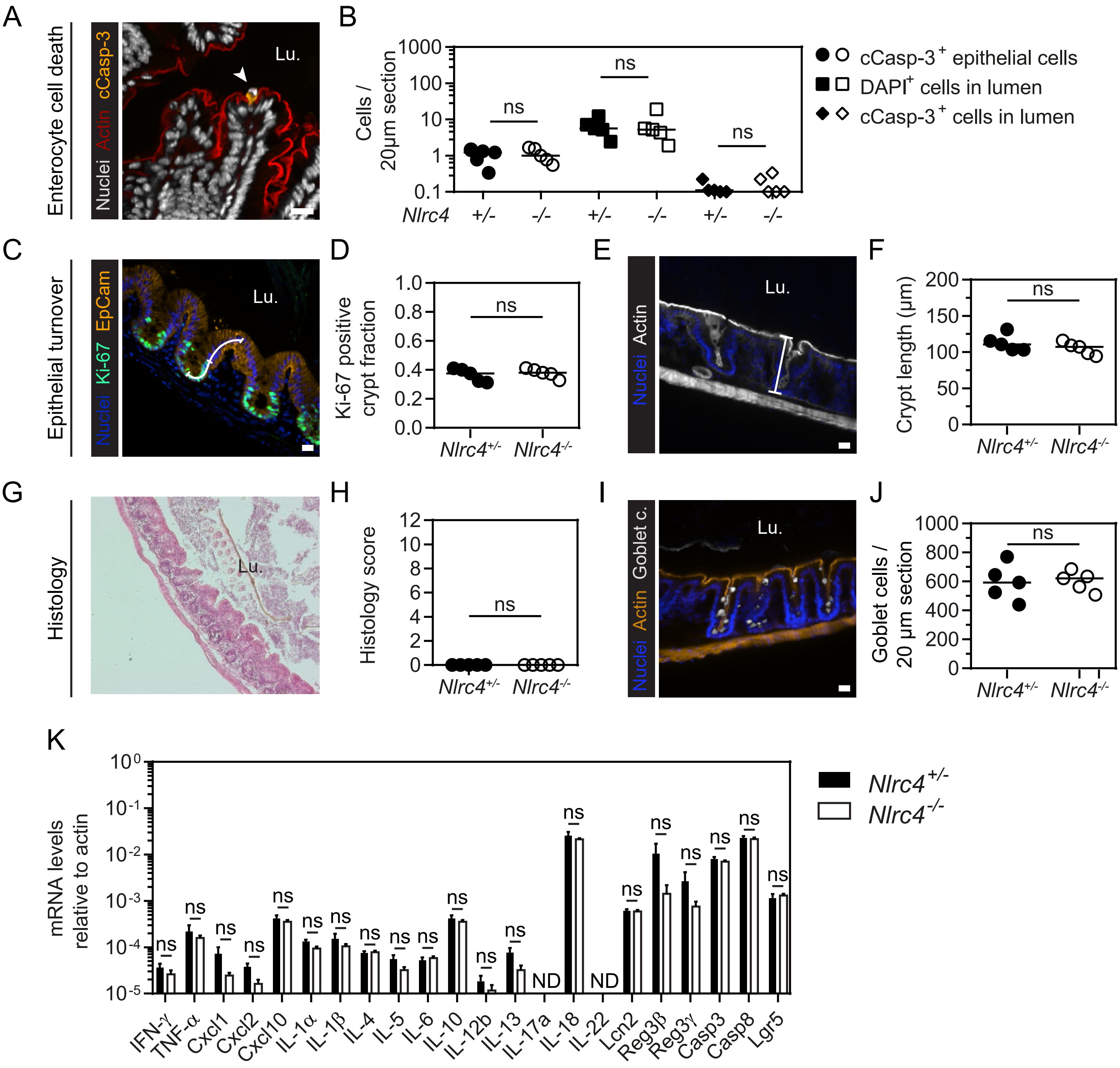

Figure S2 (steady state parameter analysis)

Steady state analysis of the cecal mucosa in *Nlrc4*$^{+/-}$ and *Nlrc4*$^{-/-}$ littermates. (**A-B**) Analysis of baseline enterocyte cell death. (**A**) Representative micrograph (from WT mouse) of a cleaved Caspase-3 positive enterocyte dislodging from epithelium. (**B**) Microscopy-based quantification of cell death. Detection limit at 0.1. (**C-F**) Analysis of epithelial turnover. (**C**) Representative micrograph (from *Nlrc4*$^{+/-}$ mouse) of crypt epithelial cell proliferation. White line indicates the way of quantification for D. (**D**) Microscopy-based quantification of Ki-67 positive crypt fraction. (**E**) Representative micrograph (from *Nlrc4*$^{-/-}$ mouse) of crypt length. White line indicates the way of quantification for F. (**F**) Microscopy-based quantification of crypt length. (**G-J**) Analysis of steady state inflammation parameters. (**G**) Representative micrograph (from *Nlrc4*$^{-/-}$ mouse) of a paraffin-embedded H&E-stained cecal tissue section. (**H**) Microscopy-based quantification of histology score. (**I**) Representative micrograph (from *Nlrc4*$^{-/-}$ mouse) of goblet cells. (**J**) Microscopy-based quantification of goblet cells per 20µm section. (**K**) mRNA levels of pro-inflammatory cytokines, anti-microbial peptides, Caspases and Lgr5. Means with SD are indicated. Data from 5 *Nlrc4*$^{+/-}$ and 6 *Nlrc4*$^{-/-}$ mice. In B, D, F, H and J each data point represents one mouse. Line at median. 5 mice per group. In A, C, E and I scale bars: 20µm. Lu. - Lumen. In B, D, F, H and J Mann-Whitney U-test. K Multiple t-test using Bonferroni-Dunn method (ns - not significant).

Figure S3 - Supplementary figure to Fig2

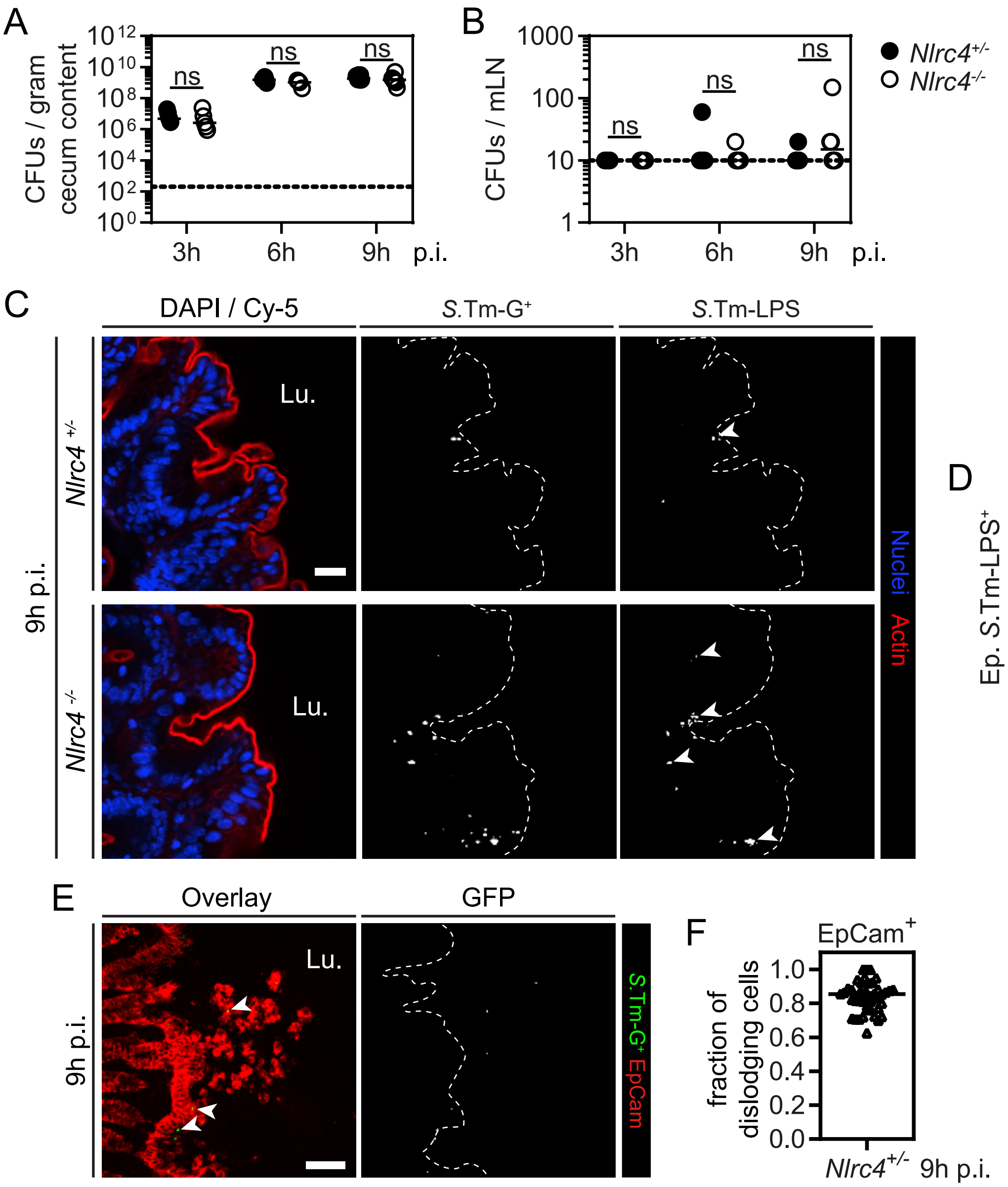

Figure S3 (supporting data for Figure 2)

(**A-B**) *S.*Tm CFU counts over time in *Nlrc4*$^{+/-}$ and *Nlrc4*$^{-/-}$ littermates. (**A**) *S.*Tm CFU counts in cecum content. (**B**) *S.*Tm CFU counts in mesenteric lymph nodes. In A-B dashed lines represent detection limit. (**C-D**) Validation of reporter plasmid *pssaG*-GFPmut2. (**C**) Representative micrographs of epithelial *S.*Tm/*pssaG-GFP* in *Nlrc4*$^{+/-}$ and *Nlrc4*$^{-/-}$ littermates at 9h pi., detected with either reporter plasmid, or by an antibody against *S.*Tm-LPS. Arrowheads indicate *S.*Tm-LPS^{+} cells. Scale bar: 20µm. (**D**) Microscopy-based quantification of epithelial LPS positive *S.*Tm. (**E-F**) Epithelial cell staining of *Nlrc4*$^{+/-}$ mice at 9h p.i. (**E**) Representative micrograph of dislodging enterocytes. Arrowheads indicate *S.*Tm-G^{+}. Scale bar: 50µm. (**F**) Microscopy-based quantification of EpCam positive cell fraction among dislodging cells. In A, B and D each data point represents one mouse. Line at median. In A-B 5-7 mice per group from ≥2 independent experiments for each comparison. In F each data point represents one quantified image. Line at median. In C and E Lu. - Lumen. Mann-Whitney U-test in A-B (ns - not significant).

Figure S4 - Supplementary figure to Fig3

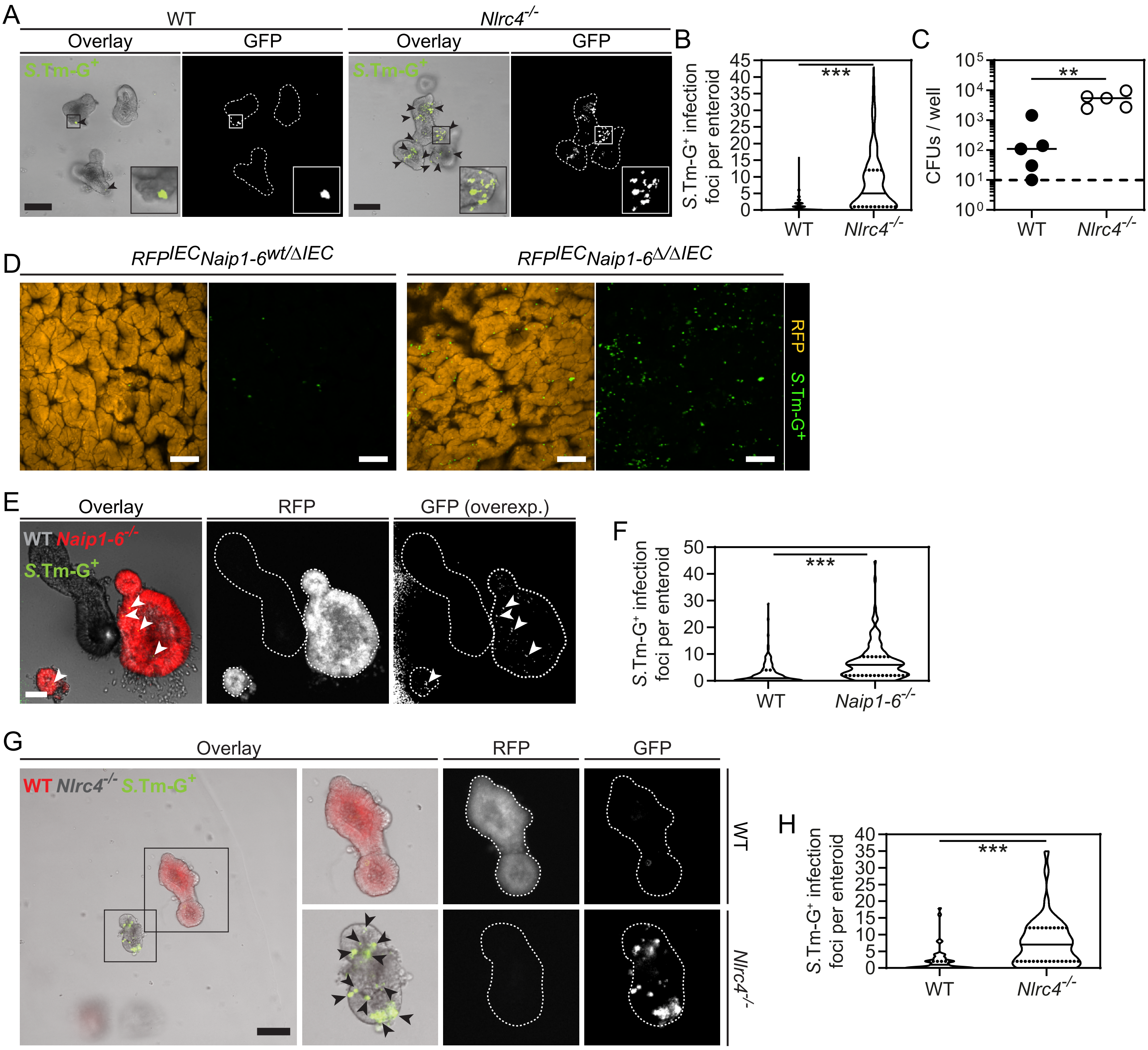

Figure S4 (supporting data for Figure 3)

(**A-H**) The *in vivo* NAIP/NLRC4 phenotype of high epithelial *S.*Tm loads can be observed also in cultured enteroids. (**A**) Representative micrographs of *S.*Tm/*pssaG-GFP*-infected WT and $Nlrc4^{-/-}$ enteroids (MOI 100) at 4h p.i. Arrowheads indicate intracellular *S.*Tm-G^{+} infection foci. Scale bar: 100µm. (**B**) Microscopy-based quantification of intracellular *S.*Tm-G^{+} infection foci per enteroid. (**C**) *S.*Tm CFU counts per well of WT and $Nlrc4^{-/-}$ enteroids at 21h p.i. Each data point represents one well and line at median. Dashed line represents detection limit. (**D**) Representative micrographs of RFP-expressing cecal tissue explants at 18h p.i. of $RFP^{IEC}Naip1\text{-}6^{wt/\Delta IEC}$ and $RFP^{IEC}Naip1\text{-}6^{\Delta/\Delta IEC}$ littermates. Scale bars: 100µm. (**E**) Representative micrographs of *S.*Tm/*pssaG-GFP*-infected (MOI 100) WT and RFP-expressing NAIP1-6-deficient mixed enteroid cultures at 4h p.i. Arrowheads indicate intracellular *S.*Tm-G^{+} infection foci. Third panel represents an overexposure to make *S.*Tm-G^{+} infection foci visible also at this low magnification. Scale bar: 50µm. (**F**) Microscopy-based quantification of intracellular *S.*Tm-G^{+} infection foci per enteroid as in E. (**G**) Representative micrographs of *S.*Tm/*pssaG-GFP*-infected (MOI 100) RFP-expressing WT and NLRC4-deficient mixed enteroid cultures at 4h p.i. Arrowheads indicate intracellular *S.*Tm-G^{+} infection foci. Scale bar: 100µm. (**H**) Microscopy-based quantification of intracellular *S.*Tm-G^{+} infection foci per enteroid as in G. In violin plots, line represents median and dashed line quartiles. Data in B, F and H are representative for 3 replicate infections, data in C for 2 replicate infections. In B, C, F and H Mann-Whitney U-test (**p<0.01, ***p<0.001).

Figure S5 - Supplementary figure to Fig3

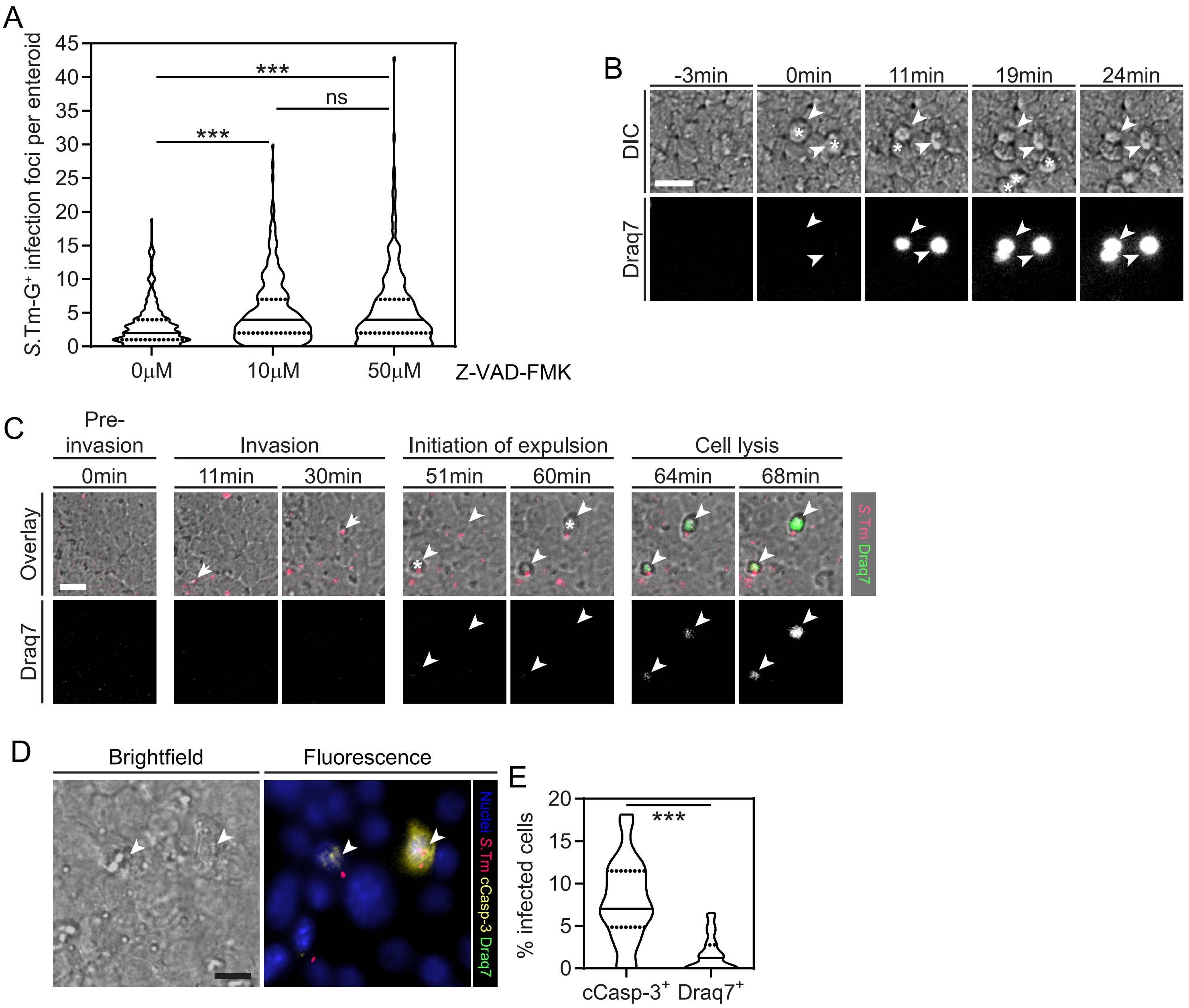

Figure S5 (supporting data for Figure 3)

(**A**) Microscopy-based quantification of intracellular *S.*Tm-G^{+} infection foci in WT enteroids treated with different concentrations of pan-caspase inhibitor Z-VAD-FMK, infected at MOI 100, and analyzed at 4h p.i. (**B**) An additional representative time-lapse microscopy image series of *S.*Tm-infected 2D enteroid monolayers (MOI 2). Cell lysis was determined using membrane impermeable nuclear dye Draq7. Examples of expelling enterocytes indicated by arrowheads - time point 0 represents the first sign of expulsion of the two indicated cells, as judged from DIC microscopy. Stars represent first sign of expulsion. Scale bar: 20µm. (**C**) Representative time-lapse microscopy image series of *S.*Tm-mCherry-infected 2D enteroid monolayers (MOI 2). Cell lysis was determined using membrane impermeable nuclear dye Draq7. Examples of *S.*Tm invasion indicated by arrows. Examples of expelling infected enterocytes indicated by arrowheads, as judged from DIC microscopy. Stars represent first sign of expulsion. Scale bar: 20µm. (**D-E**) Fixed and cleaved Caspase-3 stained 2D enteroid monolayers subsequent to infection with *S.*Tm-mCherry (MOI 2) in the presence of Draq7. (**D**) Representative micrographs. Arrowheads indicate infected enterocytes staining partially or completely positive for cleaved Caspase-3. Scale bar: 10µm. (**E**) Microscopy-based quantification of cleaved Caspase-3^{+} and Draq7^{+} infected enterocytes. In violin plots, line represents median and dashed line quartiles. Data in A and E are representative for ≥2 infections. In A two-way ANOVA with Tukey HSD and in E Mann-Whitney U-test (ns - not significant, ***$p<0.001$).

Figure S6 - Supplementary figure to Fig3

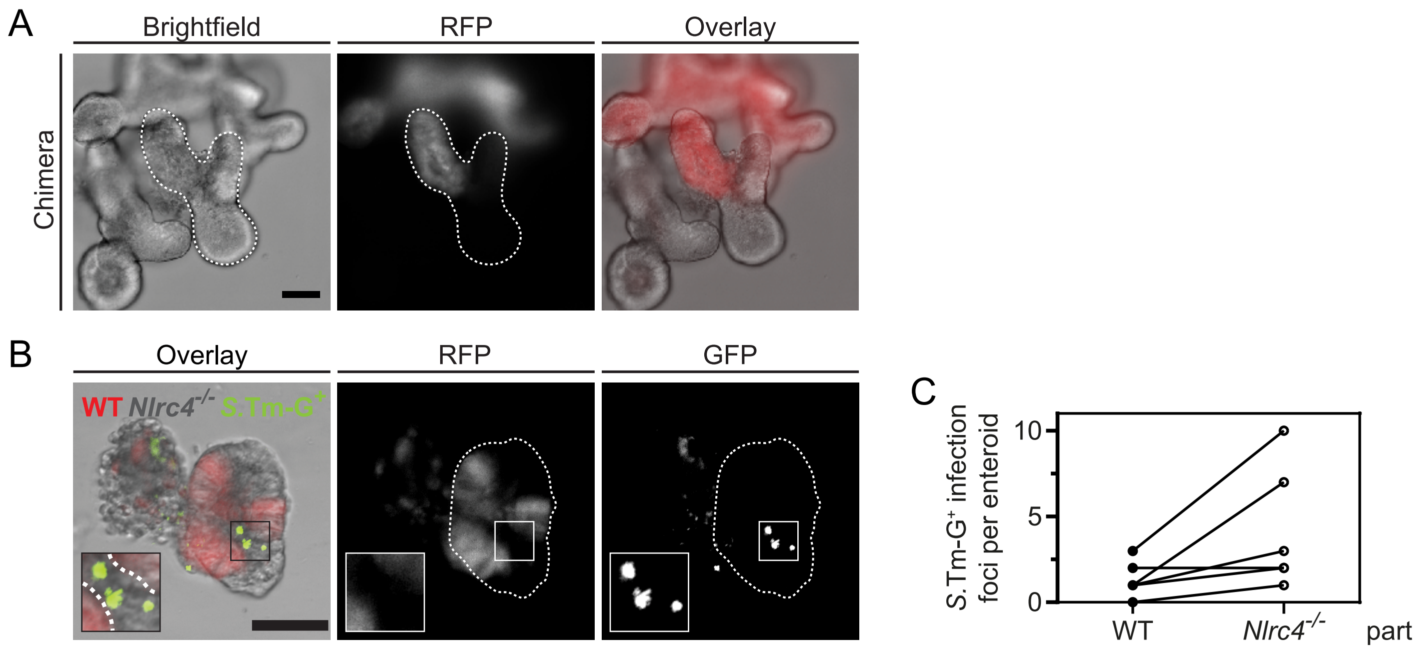

Figure S6 (supporting data for Figure 3)

(**A**) Representative micrograph of a chimeric enteroid consisting of WT and Villin-RFP WT epithelial cells. Scale bar: 50µm. (**B-C**) Chimeric 3D enteroids show higher *S.*Tm loads in NLRC4-deficient regions. (**B**) Representative micrographs of *S.*Tm/*pssaG-GFP*-infected (MOI 100) WT (RFP^{IEC})x$Nlrc4^{-/-}$ chimeric enteroids at 4h p.i. Scale bar: 50µm. (**C**) Microscopy-based quantification of intracellular *S.*Tm-G^{+} infection foci. Each line with connected data points represents one chimeric enteroid. B-C present data from one infection experiment (see also Fig 3E-F for supporting data).

Figure S7 - Supplementary figure to Fig4

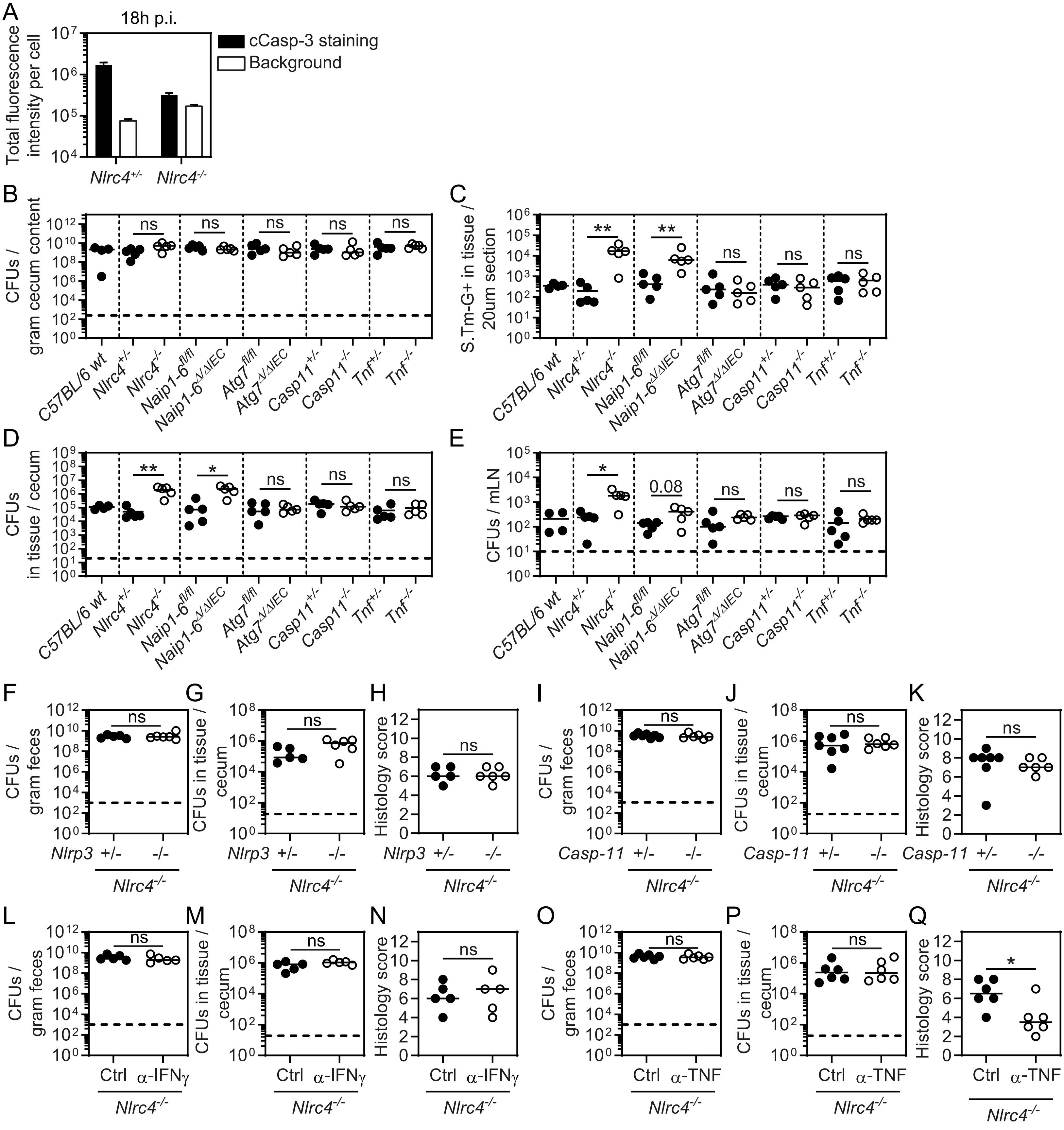

Figure S7 (supporting data for Figure 4)

(**A**) Microscopy-based quantification of total fluorescence intensity per cell from cleaved Caspase-3-stained cecal tissue sections from 5 *Nlrc4*$^{+/-}$ and 4 *Nlrc4*$^{-/-}$ littermates at 18h p.i. 4 measurements per mouse. Means with SD are indicated. (**B-E**) *S.*Tm counts in the indicated single gene knockout mice and the corresponding littermate controls at 18h p.i. (**B**) *S.*Tm CFU counts in cecum content. (**C**) Microscopy-based quantification of *S.*Tm-G^{+} in cecal tissue. (**D**) *S.*Tm CFU counts in cecal tissue, as judged by plating of washed and homogenized tissue samples. (**E**) *S.*Tm CFU counts in mesenteric lymph nodes. (**F-H**) *S.*Tm/*pssaG-GFP* infection of *Nlrc4*$^{-/-}$*Nlrp3*$^{+/-}$ control mice and *Nlrc4*$^{-/-}$*Nlrp3*$^{-/-}$ littermates for 24h. (**F**) *S.*Tm CFU counts in feces. (**G**) *S.*Tm CFU counts in cecal tissue (**H**) Microscopy-based quantification of histology score from H&E-stained cecal tissue sections. (**I-K**) *S.*Tm/*pssaG-GFP* infection of *Nlrc4*$^{-/-}$*Caspase 11*$^{+/-}$ control mice and *Nlrc4*$^{-/-}$ *Caspase 11*$^{-/-}$ littermates for 24h. (**I**) *S.*Tm CFU counts in feces. (**J**) *S.*Tm CFU counts in cecal tissue. (**K**) Microscopy-based quantification of histology score from H&E-stained cecal tissue sections. (**L-N**) *S.*Tm/*pssaG-GFP* infection of *Nlrc4*$^{-/-}$ mice for 24h, injected (i.p.) with anti-IFNγ or isotype control antibody. (**L**) *S.*Tm CFU counts in feces. (**M**) *S.*Tm CFU counts in cecal tissue. (**N**) Microscopy-based quantification of histology score from H&E-stained cecal tissue sections. (**O-Q**) *S.*Tm/*pssaG-GFP* infection of *Nlrc4*$^{-/-}$ mice for 24h, injected (i.p.) with anti-TNF or isotype control antibody. (**O**) *S.*Tm CFU counts in feces. (**P**) *S.*Tm CFU counts in cecal tissue. (**Q**) Microscopy-based quantification of histology score from H&E-stained cecal tissue sections. In B-Q each data point represents one mouse. Line at median. Dashed lines represent detection limit. 5-7 mice per group from ≥2 independent experiments for each comparison (except WT ctrl in B-E, 4 mice). Mann-Whitney U-test (ns - not significant, *p<0.05, **p<0.01).

Figure S8 - Supplementary figure to Fig5

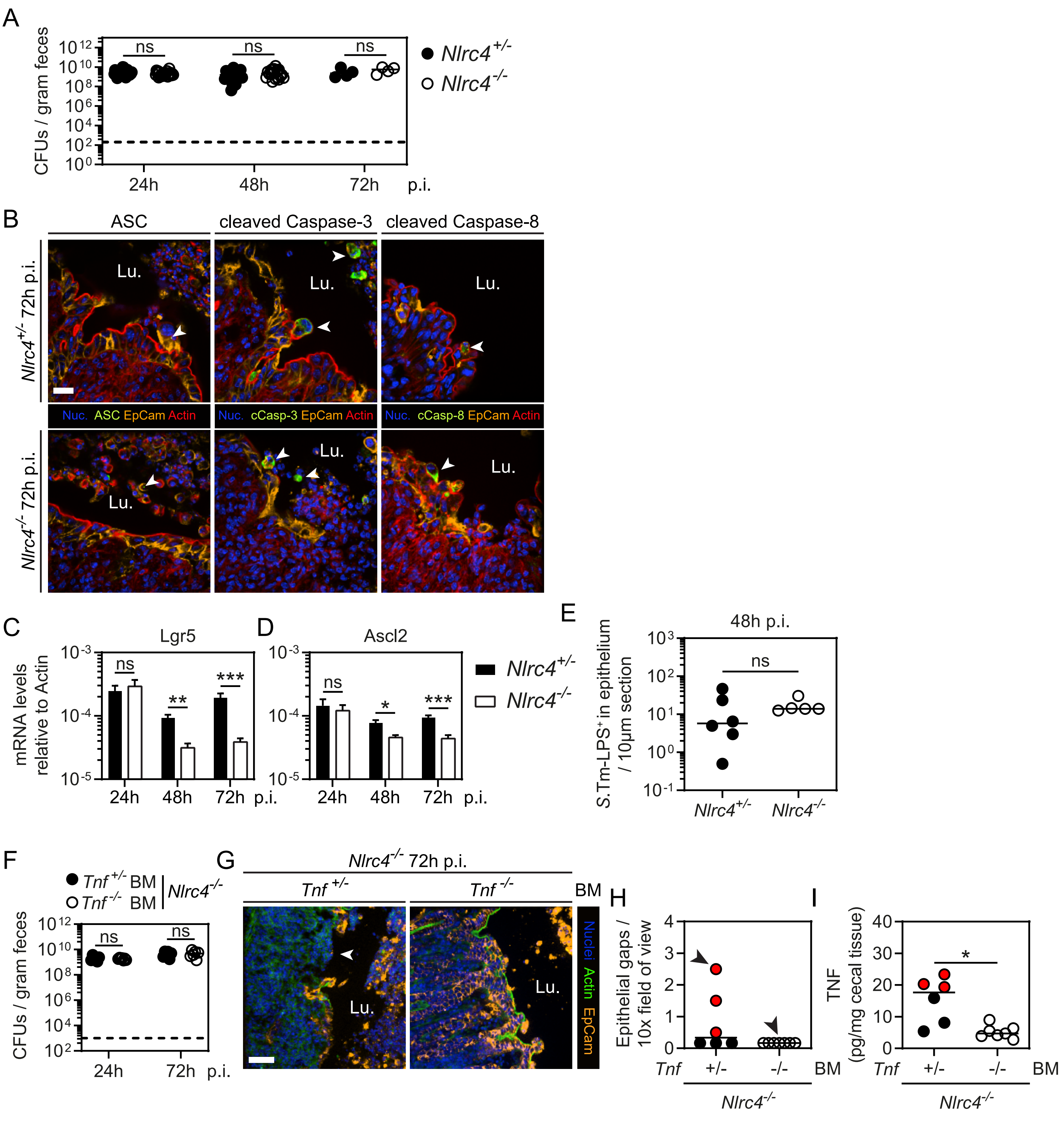

Figure S8 (supporting data for Figure 5)

(**A**) *S.*Tm CFU counts in feces over time in *Nlrc4*$^{+/-}$ and *Nlrc4*$^{-/-}$ littermates. Each data point represents one mouse. Line at median. Dashed line indicates detection limit. (**B**) Micrographs of cecal tissue from *Nlrc4*$^{+/-}$ and *Nlrc4*$^{-/-}$ littermates at 72h p.i. stained for ASC, cleaved Caspase-3 and cleaved Caspase-8. White arrowheads indicate dislodging/dislodged cells. Lu. - Lumen. Scale bar: 20µm. (**C-D**) Cecal tissue mRNA levels of stem cell markers over time in *Nlrc4*$^{+/-}$ and *Nlrc4*$^{-/-}$ littermates. (**C**) mRNA level of Lgr5. (**D**) mRNA levels of Ascl2. Means and SD are indicated. In A, C-D 5-10 mice per group from ≥2 independent experiments per comparison (In A at 72h only 4 mice gave fecal pellets). (**E**) Microscopy-based quantification of cecal tissue from *Nlrc4*$^{+/-}$ and *Nlrc4*$^{-/-}$ littermates at 48h p.i. for epithelial *S.*Tm-LPS^{+} cells. (**F-I**) Infection of bone marrow chimeras for 72h. (**F**) *S.*Tm CFU counts in feces. Dashed line indicates detection limit. (**G-I**) Epithelial gaps correlate with high TNF levels. (**G**) Micrograph of cecal tissue from mice indicated with arrowheads in H. White arrowheads indicate epithelial gaps. Lu. - Lumen. Scale bar: 50µm. (**H**) Microscopy-based quantification of epithelial gaps per 10x field of view. Arrowheads indicate mice shown in G. Red dots represents the same mice as red dots in I. Lowest dots at detection limit of 0.17. (**I**) TNF concentrations in cecal tissue. Same figure as Fig 5I, but with red dots highlighted, representing the same mice as in H. In E-I 5-7 mice per group from ≥2 independent experiments per comparison. In A, C-F, H-I Mann-Whitney U-test (ns - not significant, *p<0.05, **p<0.01, ***p<0.001).

Figure S9 - Supplementary figure to Fig6

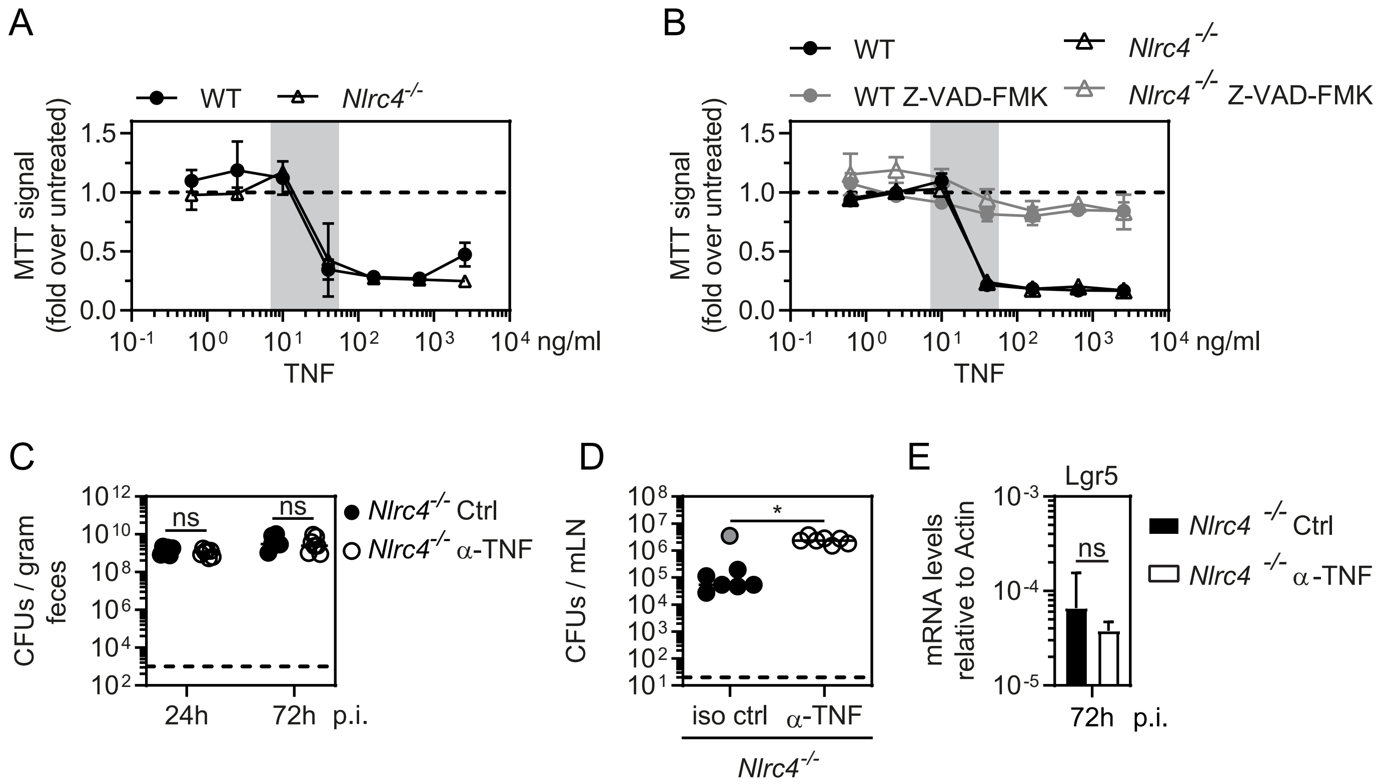

Figure S9 (supporting data for Figure 6)

(**A**) WT and *Nlrc4*$^{-/-}$ enteroids respond similarly to TNF. Enteroids with the indicated genotypes were exposed in parallel to TNF directly after seeding. MTT assay at 10 days post-seeding. Fold change of light absorption at 562nm over untreated is plotted. (**B**) TNF effect disappears in the presence of pan-caspase inhibitor Z-VAD-FMK. Enteroids with the indicated genotypes were exposed in parallel to TNF in the absence or presence of 50µM Z-VAD-FMK directly after seeding. MTT assay at 10 days post-seeding. Fold change of light absorption at 562nm over untreated is plotted. In A-B, each data point represents the means with SD of ≥2 separately treated cultures. Data are representative for ≥2 separate experiments. In A-B grey area represents the approximate range of TNF concentrations measured in littermate controls and *Nlrc4*$^{-/-}$ mice (derived from data in Fig 5F). (**C-E**) 72h *S.*Tm infection of *Nlrc4*$^{-/-}$ mice treated with isotype control or anti-TNF. (**C**) *S.*Tm CFU counts in feces (**D**) *S.*Tm CFU counts in mesenteric lymph nodes. Dashed line indicates detection limit. Each data point represents one mouse. Line at median. 7 mice per group from 2 independent experiments. Grey dot indicates a suspected outlier mouse that showed early signs of severe systemic infection (ruffled fur, hunched back etc.) normally not noted under these conditions, but which can result from esophageal damage during per-oral gavage. (**E**) Cecal tissue mRNA levels of stem cell marker Lgr5. Means and SD are indicated. In C-E Mann-Whitney U-test (ns - not significant, **p<0.01).

Figure S10 - Conceptual model

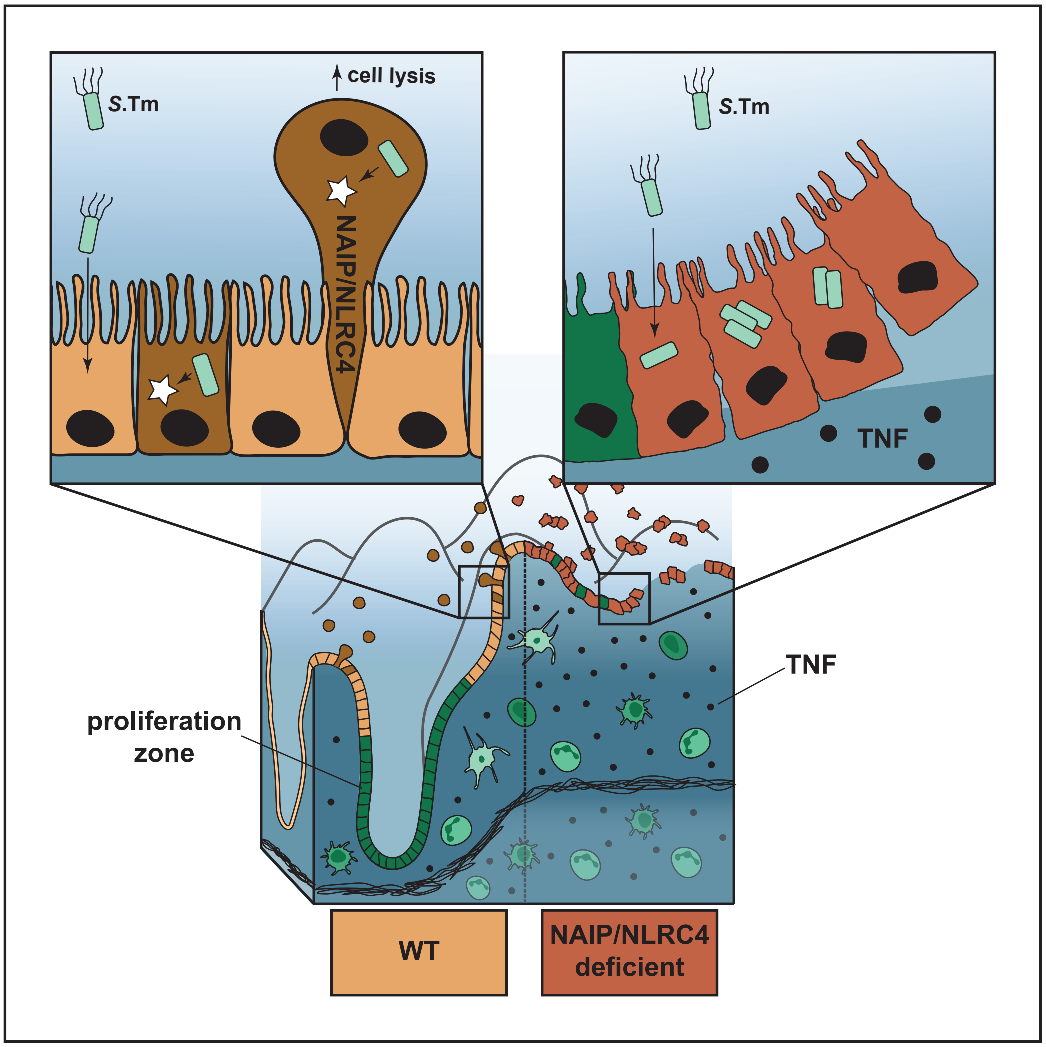

Figure S10 (Conceptual model)
